# Transcriptome analysis of the effect of pyrroloquinoline quinone disodium (PQQ·Na_2_) on reproductive performance in sows during gestation and lactation

**DOI:** 10.1186/s40104-019-0369-y

**Published:** 2019-08-07

**Authors:** Boru Zhang, Chenxi Wang, Wei Yang, Hongyun Zhang, Qingwei Meng, Baoming Shi, Anshan Shan

**Affiliations:** Institute of Animal Nutrition, Northeast Agricultural University, Harbin, 150030 People’s Republic of China

**Keywords:** Oxidative stress, Pyrroloquinoline quinone, Pyrroloquinoline quinone disodium, Reproductive performance, RNA-seq, Sow

## Abstract

**Background:**

Pyrroloquinoline quinone (PQQ), which is a water soluble, thermo-stable triglyceride-quinone, is widely distributed in nature and characterized as a mammalian vitamin-like redox cofactor. The objective of this study was to investigate the effects of pyrroloquinoline quinone disodium (PQQ·Na_2_) on reproductive performance in sows.

**Results:**

Dietary supplementation with PQQ·Na_2_ significantly increased the total number of piglets born, the number of piglets born alive and the born alive litter weight. It also increased the antioxidant status in the placenta, plasma and milk. The concentration of NO was significantly increased in the plasma and placenta. RNA-seq analysis showed that 462 unigenes were differentially expressed between the control (Con) treatment and PQQ treatment groups. Among these unigenes, 199 were upregulated, while 263 unigenes were downregulated. The assigned functions of the unigenes covered a broad range of GO categories. Reproduction (27, 7.03%) and the reproduction process (27, 7.03%) were assigned to the biological process category. By matching DEGs to the KEGG database, we identified 29 pathways.

**Conclusions:**

In conclusion, dietary supplementation with PQQ·Na_2_ in gestating and lactating sows had positive effects on their reproductive performance.

**Electronic supplementary material:**

The online version of this article (10.1186/s40104-019-0369-y) contains supplementary material, which is available to authorized users.

## Background

Sow reproductive performance affects the production level and provides an economic benefit to the pig industry. Litter size is an important reproductive trait and a critical indicator of sow reproduction performance [1]. Maintaining optimal reproductive and litter performance is essential for meeting economic targets in commercial pig production [2]. Increasing litter size has long been a goal of pig breeders and producers; prolificacy is of great interest to the pig industry [3]. Maternal nutrition has substantial implications for fetal health. Enhancing reproductive performance through nutrition and management strategies in gestation and lactation sows have been of research interest for several decades [4]. The placental tissue is the only site for contact between the fetus and the mother during pregnancy; thus, the tissue is closely related to the health and development of the fetus [5]. Maternal conditions have been demonstrated to affect the placental morphology, blood flow, fetomaternal exchanges, and endocrine function [6] . In addition, the placenta plays a pivotal role in maternal nutrient supply and metabolic waste removal, protection against bacterial and viral infections, and production of hormones supporting pregnancy [7].

Pyrroloquinoline quinone (PQQ), which is a water soluble, thermo-stable triglyceride-quinone [8]. Initially identified as a novel cofactor of various bacterial dehydrogenases [9], PQQ is an essential animal nutrient. PQQ-deficient animals display a variety of illnesses [10]. PQQ has attracted considerable attention, as it is important for mammalian growth, development, reproduction and immune function [11]. PQQ is an effective antioxidant, protecting mitochondria against oxidative stress-induced lipid peroxidation, protein carbonyl formation and inactivation of the mitochondrial respiratory chain [12]. Although PQQ is not biosynthesized in mammals, trace amounts of PQQ have been found in human and rat tissues at picomolar to nanomolar levels, and an especially large amount has been found in human milk [13].

Because of its versatile functions, PQQ disodium (PQQ·Na_2_) salt has been authorized in Canada as a Natural Health Product, providing 20 mg PQQ·Na_2_ salt per day as an antioxidant for the maintenance of good health [14]. On August 13, 2018, the European Commission issued regulations (EU) 2018/1122 approving pyrroquinone sodium salt as a new type of food. The European commission defines pyrroquinone sodium as a dietary supplement. Although PQQ has a positive effect on reproductive performance, its mechanism is not clear. Therefore, in this study, we used the Illumina HiSeqTM2500 platform to perform a large-scale transcriptome analysis of the placenta of sows. The next-generation RNA sequencing (RNA-seq) platform has emerged as the method of choice for studying transcriptomes [15]. Second generation sequencing technology, also called RNA sequencing (RNA-seq), is powerful for gene identification, comparative gene expression analysis and investigation of the functional complexity of the transcriptome [16]. In recent years, an RNA-seq approach has been widely used in animals for novel gene identification and differentially expressed gene (DEGs) analysis, because it is high throughput, low cost, covers a multitude of low abundance genes, and has high sensitivity. Although PQQ has gained interest in medicine and health-related research in recent decades, the usefulness of PQQ has not yet been fully demonstrated in animal agriculture, especially in the pig industry. In addition, there are no published data on the effects of PQQ on reproductive performance in sows. Therefore, the objective of this study was to test the efficacy of dietary PQQ·Na_2_ supplementation on reproductive performance. We also analyzed the RNA-seq of placentas, which revealed the genes that may be involved in placental development and function, thus playing a role in determining litter size. Furthermore, the data collected were used to establish the relationship between PQQ and the placenta of sows, as well as reproductive performance. We hope the results of this study could lay a foundation for the further study of PQQ in the pig industry.

## Methods

### Animals and management

A total of 40 crossbred (Landrace×Large White crossed with Duroc boar) multiparity gestation sows with an average parity of 4.3 were used in the study. Forty sows were allotted to 2 dietary treatments after breeding. One group was the control sows, which were fed a corn-soybean meal control diet (Con treatment, *n* = 20), and the other group was the treatment sows, fed a control diet with 20 mg/kg PQQ·Na_2_ after breeding and through gestation (PQQ treatment, *n* = 20). The PQQ·Na_2_ (purity, ≥ 98%) was synthesized by chemical reactions. It was diluted with corn starch to a concentration of 1 g/kg mixture before being mixed into the diet. Based on the known range of PQQ in foods [17], we inferred that the concentration of PQQ in the basal diet was less than 0.01 mg/kg. The sows were kept in single crates (0.6 m × 2.0 m) from insemination to day 110 of gestation. On d 110 of gestation, sows were transported to the farrowing facility, where they were placed in individual farrowing crates (2.4 m × 2.4 m). Each crate had steel mesh floors with a heat lamp for newborn pigs. The crates were mounted over a solid concrete floor, and manure was removed manually each day. The farrowing room temperature was maintained at approximately 18 to 20 °C. Births were watched, but the observers interfered as little as possible in the farrowing process. The protocols used in this experiment were approved by the Northeast Agricultural University Institutional Animal Care and Use Committee. All animal experimental diets (Table 1) were formulated to meet or exceed the recommended nutrient requirements of the NRC (2012). From d 1 of gestation until d 90 of gestation, all sows were fed 2.5 kg of the gestation diet daily. From d 91 of gestation, all sows were fed 4.5 kg of the gestation diet daily. The amounts of parturition feed provided to each sow at d 112, 113 and 114 of gestation were 2.0, 1.5 and 1.0 kg, respectively.

**Table 1:** Composition and nutrient levels of diets

Item	Gestation
Ingredients, %	
Corn	67.5
Soybean meal	16
Wheat bran	13.5
Dicalcium phosphate	1
Limestone	1.1
Salt	0.4
Premix^a^	0.5
Nutritional composition^b^, %	
Net energy, MJ/kg	9.62
Crude protein (CP)	15.58
Calcium	0.71
Total phosphorus	0.60
Available phosphorus	0.31
SID Lysine	0.54

^a^The premix provides following for per kg diet: vitamin A, 8000 U; vitamin D_3_, 2000 U; vitamin E, 50 U; vitamin K_3_, 1.5 mg; vitamin B_1_, 1.6 mg; vitamin B_6_, 1.5 mg; vitamin B_12_,15 μg; niacin, 20 mg; *D*-pantothenic acid,15 mg; Zn (ZnO), 100 mg; Fe (FeSO_4_·7H_2_O), 80 mg; Cu (CuSO_4_·5H_2_O), 20 mg; Mn (MnSO_4_·H_2_O), 25 mg; I (KI), 0.3 mg; Se (NaSeO_3_·5H_2_O), 0.2 mg

^b^Nutrient levels were calculated values

### Sample and data collection

Blood was collected from the ear vein of a random subset of sows (*n* = 8 per treatment) at d 90 of gestation and d 21 of lactation. The blood was centrifuged at 3000×*g* for 15 min to obtain the plasma, and the plasma was stored at − 20 °C until analysis. Eight sows per treatment were randomly selected and marked for milk sample collection during lactation. Colostrum was collected within 6 h of farrowing (d 0 of lactation). Approximately 30 to 50 mL of milk was collected from all functional mammary glands using a mechanical milk pump after the injection of 1 mL oxytocin. The samples were immediately stored at − 20 °C until analysis. Placenta allantochorion tissue samples were collected immediately during parturition to preserve RNA stability for mRNA analysis. A section of samples was stored at − 20 °C, and another section was snap-frozen in liquid nitrogen for further analysis. Sow back-fat thickness was measured at d 0 and 90 of gestation, within 24 h of farrowing (d 0) and d 21 of lactation (*n* = 8 per treatment). Back-fat thickness was measured at the P2 position (left side of the 10^th^ rib and 6 cm lateral to the spine) by digital B-ultrasonography (Kaixin, Xuzhou, China). At farrowing, the number of piglets born, litter birth weight and individual birth weights were recorded.

### Evaluation of antioxidant enzyme activity and the concentration of nitric oxide

Superoxide dismutase (SOD), glutathione peroxidase (GSH-Px) and catalase (CAT) enzyme activities in the milk, plasma and placenta were determined using commercially available kits (Nanjing Jiancheng Bioengineering Institute, Nanjing, China) in accordance with our previous study. The results of the measurements were expressed as U/mL in plasma and milk and as U/mg protein in placenta. Lipid peroxidation in the plasma, milk and placenta was determined by measuring the amounts of malondialdehyde (MDA) through the thiobarbituric acid method using commercially available kits (Nanjing Jiancheng Bioengineering Institute, Nanjing, China). The results of the measurements were expressed as nmol/mL in plasma and as nmol/mg protein in placenta. Nitric oxide (NO) and inducible NOS (iNOS) in the plasma and placenta of sows were determined using assay kits obtained from Jiancheng Biochemistry (Nanjing, China). The colostrum was analyzed for lactose, protein, fat, and total solids with a fully automatic milk analyzer (Milko Scan^TM^ FT+ Analyzer, Foss). The milk samples were analyzed for immunoglobulin G (IgG), immunoglobulin A (IgA), and immunoglobulin M (IgM) using immunoglobulin-specific kits (Jinma Biological Engineering Co., Ltd., Shanghai, China).

### RNA extraction, cDNA library construction and sequencing

Total RNA was isolated from the placenta using TRIzol reagent (Invitrogen, USA) according to the manufacturer’s instructions. The extracted RNA was treated with DNase I (Takara Biotechnology, China) for 45 min at 37 °C to remove residual DNA. The RNA concentration and integrity were measured using an Ultrasec^TM^ 2100 pro UV/Visible Spectrophotometer (Amersham Biosciences, Uppsala, Sweden) and gel electrophoresis. Equal amounts of high-quality RNA from each specimen were pooled for RNA-Seq library construction. A cDNA library was prepared with a TruSeq RNA sample preparation kit following the manufacturer’s instructions (Illumina) and sequenced on an Illumina HiSeq^TM^ 2500 platform in 100 pair-ended mode (Biomarker Technologies).

### *De novo* transcriptome assembly and functional annotation

To obtain clean reads, the raw reads were filtered by removing the adapter, poly-N and low-quality sequences. *De novo* assembly was performed using the Trinity method [18]. The K-mer and group pairs distance were set at 25 and 300, respectively, while the other parameters were set at default levels. Based on their overlap regions, the short reads were assembled into longer contigs, which were then clustered and further assembled into unigenes with the paired-end information. For gene functional annotation, all of the assembled transcripts were aligned to the publicly available protein databases, including the National Center for Biotechnology Information (NCBI), nonredundant protein (Nr), the Swiss-Prot protein, Gene Ontology (GO) (http://wego.genomics.org.cn/cgi-bin/wego/index.pl), Clusters of Orthologous Groups (COG), and the Kyoto Encyclopedia of Genes and Genomes (KEGG) (http://www.genome.jp/kegg/kegg2.html) using BLASTx analysis with a cut-off E-value of 10^− 5^.

### Differentially expressed genes (DEGs) analysis

Fragments per kilobase of transcript per million fragments mapped (FPKM) was calculated to represent the expression abundance of the unigenes. FPKM may reflect the molar concentration of a transcript by normalizing for RNA length and for the total read number. DEGs between PQQ treated and control samples were identified by EBSeq. An FDR (false discovery rate) < 0.05 and |fold change (FC)| ≥ 2 was set as the threshold for significantly different expression

$$ \mathrm{FPKM}=\frac{\boldsymbol{cDNA}\kern0.5em \boldsymbol{fragments}}{\boldsymbol{Mapped}\kern0.34em \boldsymbol{fragments}\left(\boldsymbol{millions}\right)\times \boldsymbol{Transcipt}\kern0.34em \boldsymbol{length}\left(\boldsymbol{kb}\right)} $$

### Quantitative real-time PCR (qRT-PCR)

Total RNA from each sample was converted into cDNA using the Prime Script RT reagent Kit (TaKaRa Bio Catalog), and the cDNA was used for qRT-PCR. GAPDH was used as an internal control gene, and it did not respond to dietary treatments. The primer sequences are shown in Table 2. qRT-PCR was performed using the SYBR Green I Kit (TaKaRa Bio Catalog). For analyses, using an ABI PRISM 7500 SDS thermal cycler, PCRs were performed with 2.0 mL of first-strand cDNA and 0.4 mL of forward and reverse primers in a final volume of 20 mL. Samples were centrifuged briefly and run on the PCR machine using the default fast program (1 cycle at 95 °C for 30 s, 40 cycles of 95 °C for 5 s and 60 °C for 34 s). All of the PCRs were performed in triplicate. The relative gene expression levels were determined using the 2^–ΔΔCt^ method.

**Table 2:** Primers used for Real-time PCR

Genes	Primer sequence (5′→3′)	Product size, bp	GenBank No.
*GAPDH*	F: ATGGTGAAGGTCGGAGTGAA R: CCGTGGGTGGAATCATACTG	155	NM_001206359.1
*SOD1*	F: TCCATGTCCATCAGTTTGGA R: AGTCACATTGCCCAGGTCTC	131	NM_001190422.1
*IL-6*	F: AGCAAGGAGGTACTGGCAGA R: GTGGTGGCTTTGTCTGGATT	257	NM_001252429.1
*IL-8*	F: ACTTCCAAACTGGCTGTTGC R:GGAATGCGTATTTATGCACTGG	120	NM_213867.1
*NOS2*	F: CGTTATGCCACCAACAATGG R: GTGCCATCAGGCATCTGGTA	134	NM_001143690.1
*CDX2*	F: GTCGCTACATCACCATTCGG R: GATTTTCCTCTCCTTCGCTCT	110	NM_001278769.1
*CCN1*	F: TCGGCAGCCTGAAAA AGGGCA R: TCGCAGCGGAAGCGCATCTT	122	NM_001012022.1
*GCLC*	F: GCATGTGGCTCACCTCTTCATCAG R: GGAGGCTTGAATCTCATCGTCTGC	135	XM_021098556.1
*CALM*	F: GCTCATCGCCAGAGTGGACAAG R: GCCTGCATCACCGTGACCATG	84	XM_005668226.3

*IL-6* interleukin 6; *IL-8* interleukin 8; *SOD1* superoxidedismutase 1; *NOS2* nitric oxide synthase 2; *CDX2* caudal type homeobox 2; *CCN1* cellular communication network factor 1; *GCLC* glutamate-cysteine ligase catalytic subunit; *CALM* calmodulin

### Statistical analysis

All data analyses were performed with SPSS 19.0 software (IBM-SPSS Inc., Chicago, Illinois, USA). The data were analyzed by One-way analysis of variance (ANOVA), and multiple comparisons were analyzed with the Tukey’s test in SPSS. The individual sow and her litter were defined as the experimental unit. The results were presented as the mean values and the standard error of the mean (SEM). In all statistical tests used, *P* < 0.05 was considered significantly different.

## Results

### Reproductive performance

The results pertaining to the reproductive performance of sows are shown in Table 3. The back-fat thickness of sows at d 0 and 90 of gestation and at weaning did not differ between the two dietary treatments (*P* > 0.05). Dietary PQQ·Na_2_ supplementation in the gestation and lactation diets had no effect on back-fat thickness gain during gestation or loss during lactation (*P* > 0.05). The total piglets born, number of piglets born alive and born alive litter weight were significantly increased (*P* < 0.05) by dietary PQQ·Na_2_ supplementation during gestation and lactation. The number of piglets weaned, litter weaning weight and piglet weaning weight were not affected by dietary PQQ·Na_2_ during gestation and lactation. (*P* > 0.05).

**Table 3:** Effects of dietary PQQ·Na_2_ on reproductive performance of sows

Item	Con		PQQ		*P*-value
	Mean	SEM	Mean	SEM	
Reproductive performance					
Total piglets born	11.53^b^	0.42	12.93^a^	0.46	0.024
Number of piglets born alive	10.67^b^	0.32	11.87^a^	0.37	0.022
Born alive litter weight, kg	15.58^b^	0.57	17.50^a^	0.64	0.036
Number of piglets weaned	9.60	0.31	10.2	0.41	0.260
Litter weaning weight, kg	52.16	1.60	55.75	1.79	0.152
Piglet weaning weight, kg	5.43	0.13	5.47	0.19	0.791
Sow back-fat thickness, mm					
Gestation (d 0)	14.29	0.44	14.22	0.31	0.903
Gestation (d 90)	15.77	0.30	16.13	0.38	0.475
Gain	1.49	0.23	1.91	0.32	0.393
Parturition	17.36	0.28	17.57	0.34	0.602
Weaning	15.81	0.24	15.80	0.19	0.956
Loss	1.55	0.20	1.77	0.30	0.547

^ab^ Within a row, means without a common superscript differ (*P* < 0.05) *n* = 15. SEM, Standard error of the mean

### Antioxidant status and the concentration of nitric oxide

**Fig. 1:**
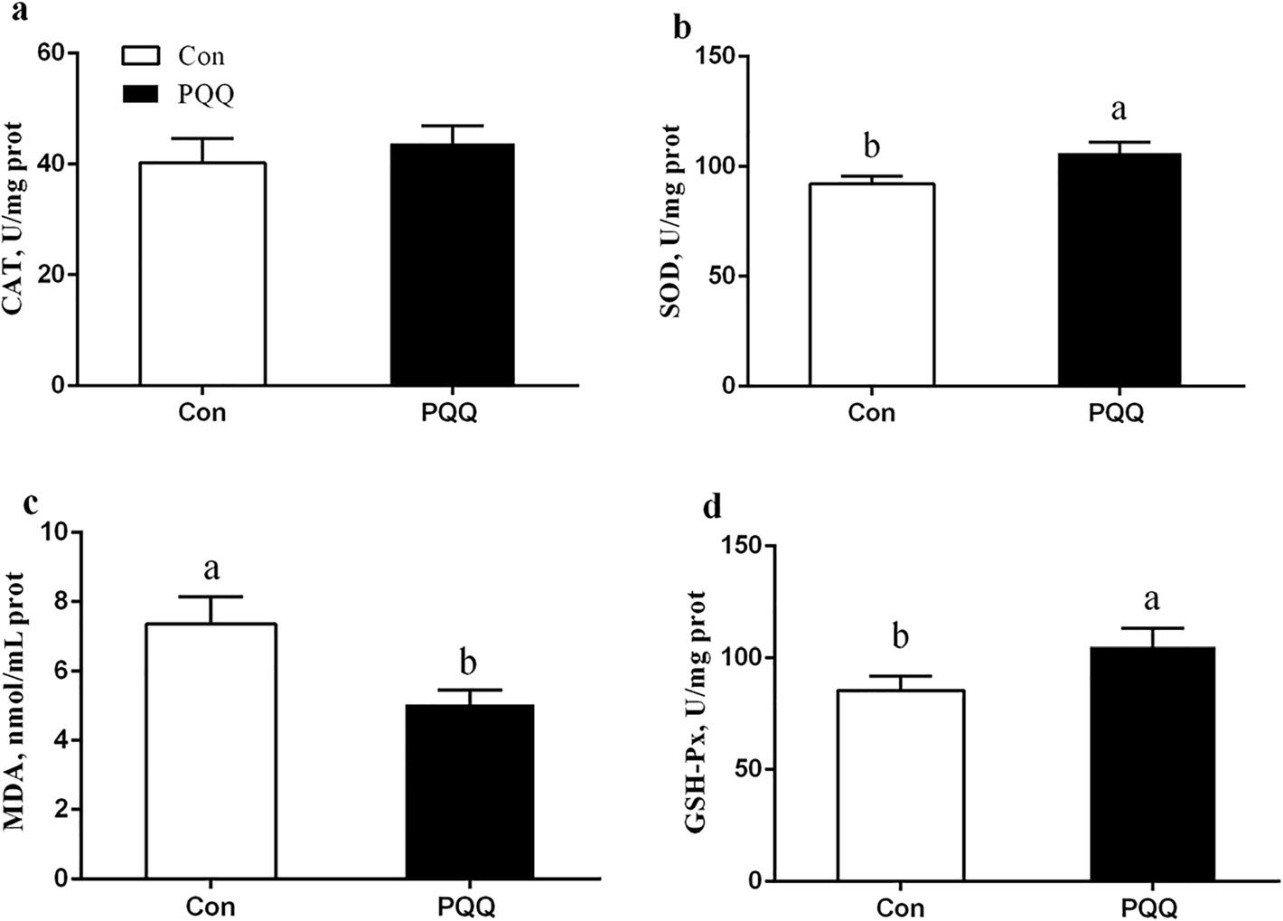
Effects of dietary PQQ·Na_2_ supplementation during gestation and lactation on antioxidant status in placenta of sows. Con, control treatment; PQQ, PQQ·Na_2_ treatment; **a** CAT, catalase; **b** SOD, superoxide dismutase; **c** MDA, malondialdehyde; **d** GSH-Px, glutathione peroxidase. All values are expressed as means ± SEM (*n* = 8). a, b Mean values within a column with unlike superscript letters were significantly different (*P* < 0.05)

**Fig. 2:**
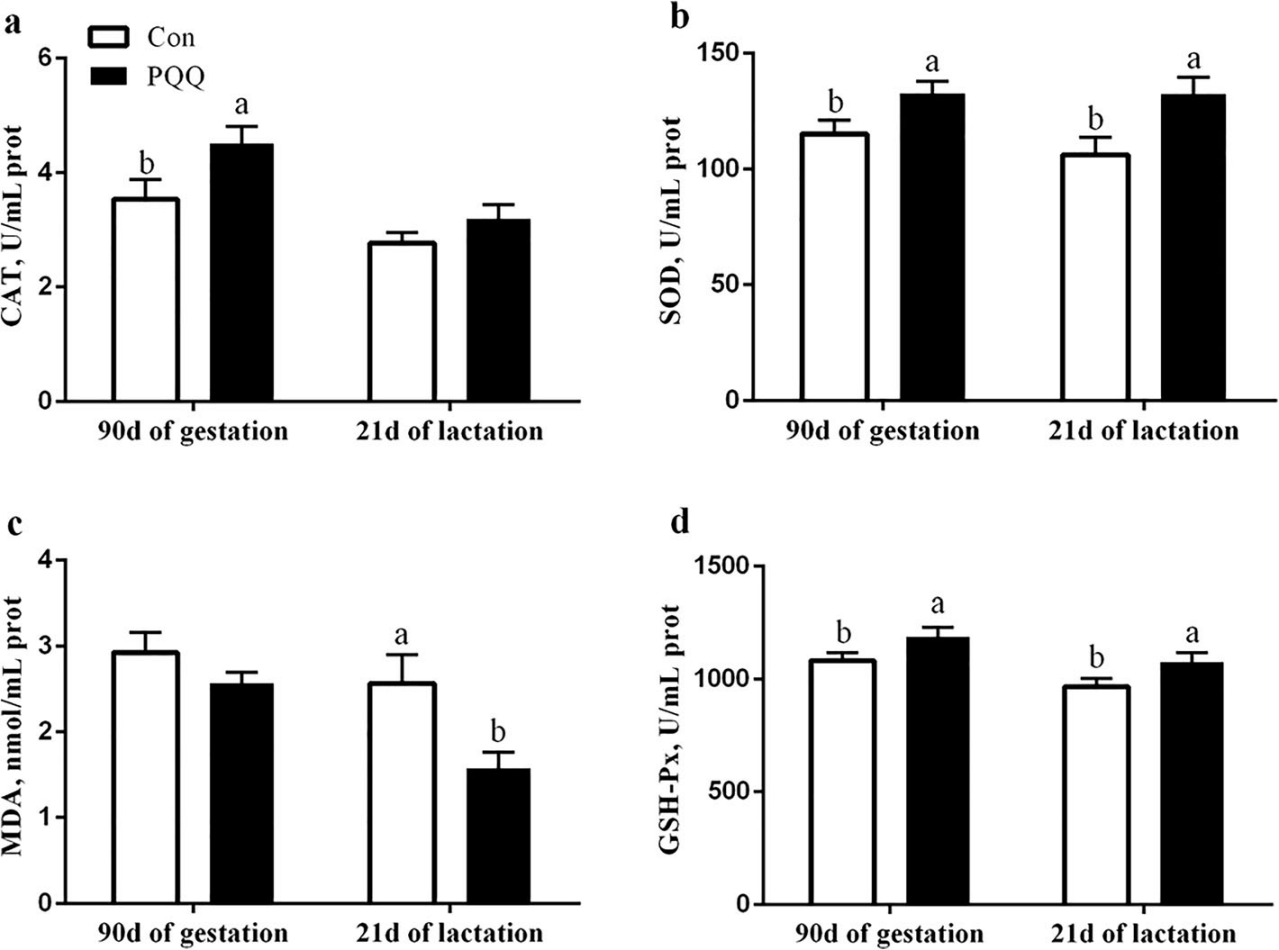
Effects of dietary PQQ·Na_2_ supplementation during gestation and lactation on antioxidant status in plasma of sows. Con, control treatment; PQQ, PQQ·Na_2_ treatment; **a** CAT, catalase; **b** SOD, superoxide dismutase; **c** MDA, malondialdehyde; **d** GSH-Px, glutathione peroxidase. All values are expressed as means ± SEM (*n* = 8). a, b Mean values within a column with unlike superscript letters were significantly different (*P* < 0.05)

**Fig. 3:**
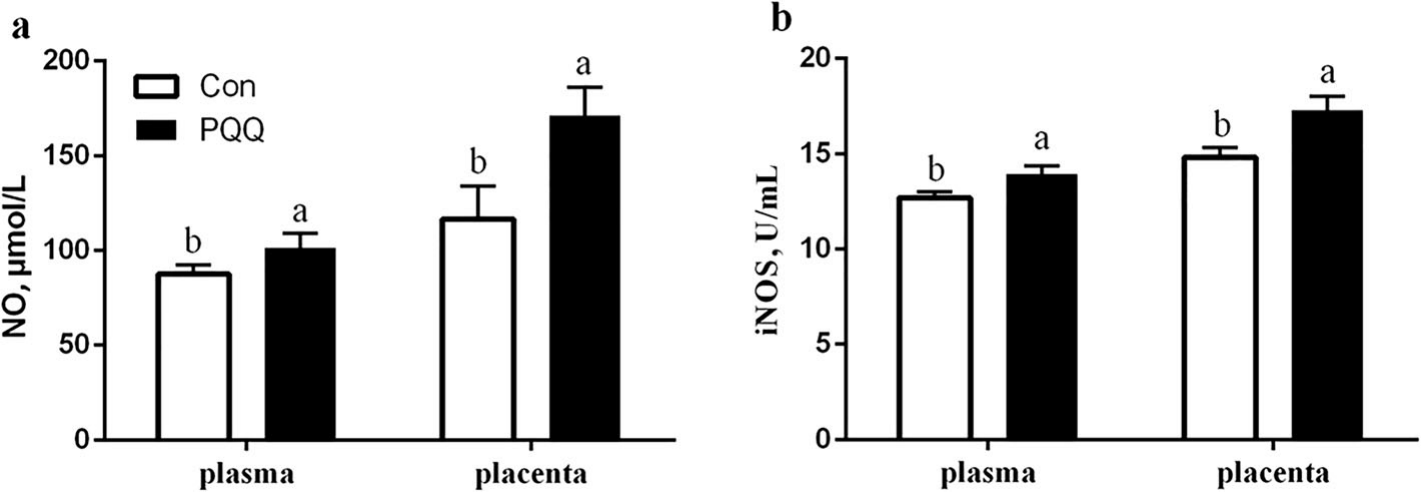
Effects of dietary PQQ·Na_2_ supplementation during gestation and lactation on the concentration of nitric oxide and inducible NOS in plasma and placenta of sows. Con, control treatment; PQQ, PQQ·Na_2_ treatment; **a** NO, nitric oxide; **b** iNOS, inducible NOS. All values are expressed as means ± SEM (*n *= 8). a,b Mean values within a column with unlike superscript letters were significantly different (*P* < 0.05)

The effects of dietary PQQ·Na_2_ supplementation during gestation and lactation on the antioxidant capacity in the placenta of sows are shown in Fig. 1. PQQ·Na_2_ dietary supplementation in gestation and lactation significantly increased the activities of SOD (*P* = 0.011) and GSH-Px (*P* = 0.032). The MDA (*P* = 0.018) activity in the placenta after PQQ·Na_2_ supplementation was significantly lower than the control treatment. However, the CAT activity of the placenta was not significantly different (*P* > 0.05). The results of the antioxidant status in the plasma of sows are presented in Fig. 2. On d 90 of gestation, the activities of CAT (*P* = 0.029), SOD (*P* = 0.041) and GSH-Px (*P* = 0.030) were significantly increased by PQQ·Na_2_ supplementation. The MDA (*P* > 0.05) activity of the placenta was not significantly changed. On d 21 of lactation, the SOD (*P* = 0.027) and GSH-Px (*P* = 0.023) activities were significantly increased and the MDA (*P* = 0.023) activity was significantly decreased by PQQ·Na_2_ supplementation. The concentration of NO (*P* = 0.020) and iNOS (*P* = 0.037) were significantly increased in plasma on d 90 of gestation by PQQ·Na_2_ supplementation are shown in Fig. 3. PQQ·Na_2_ dietary supplementation in gestation and lactation significantly increased the concentration of NO (*P* = 0.034) and iNOS (*P* = 0.016) in the placenta of sows.

### The colostrum

**Fig. 4:**
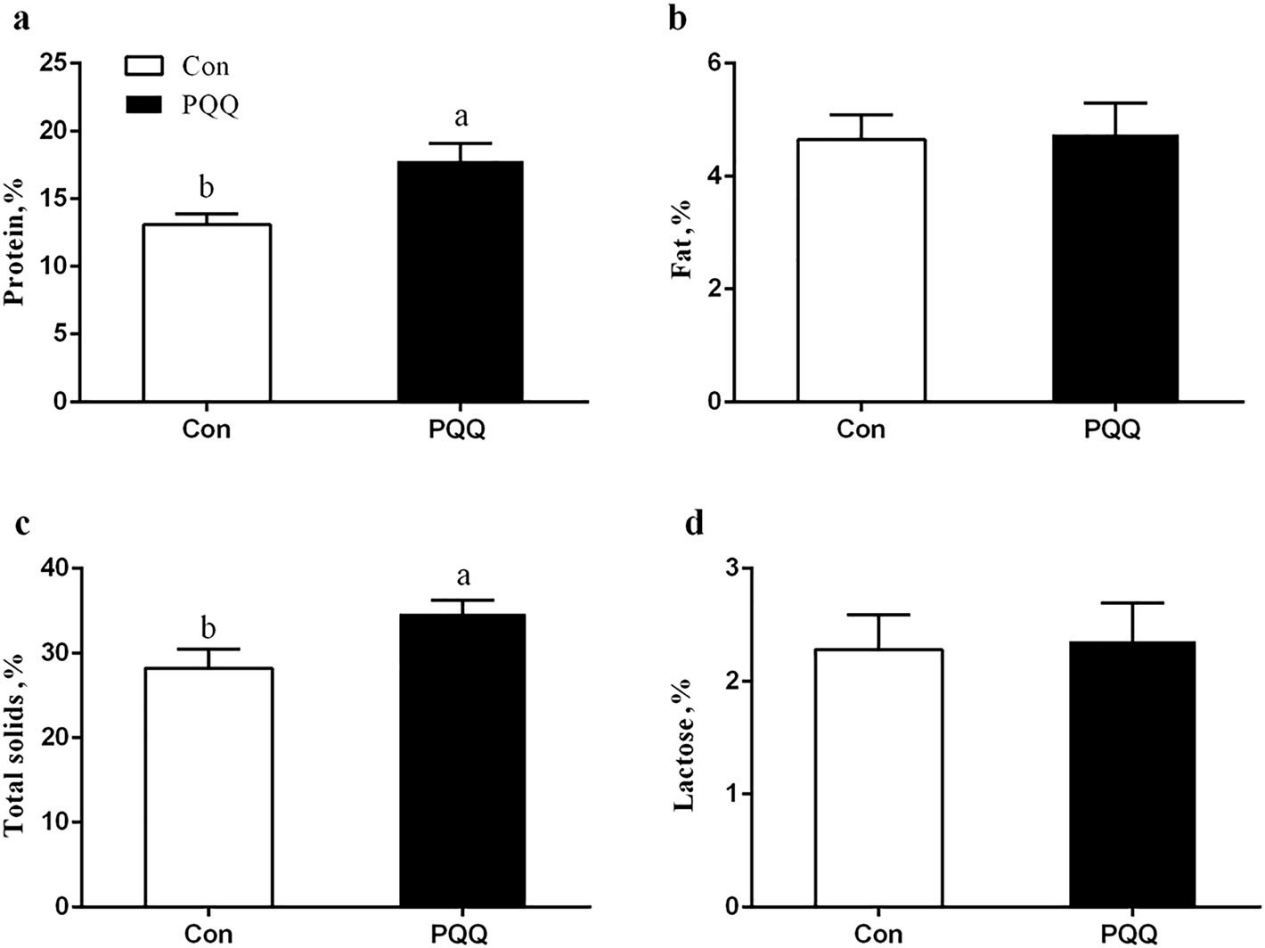
Effects of dietary PQQ·Na_2_ supplementation during gestation and lactation on the colostrum analysis in milk. **a** protein; **b** fat; **c** total solids; d lactose. All values are expressed as means ± SEM (*n* = 6). a, b Mean values within a column with unlike superscript letters were significantly different (*P* < 0.05)

**Fig. 5:**
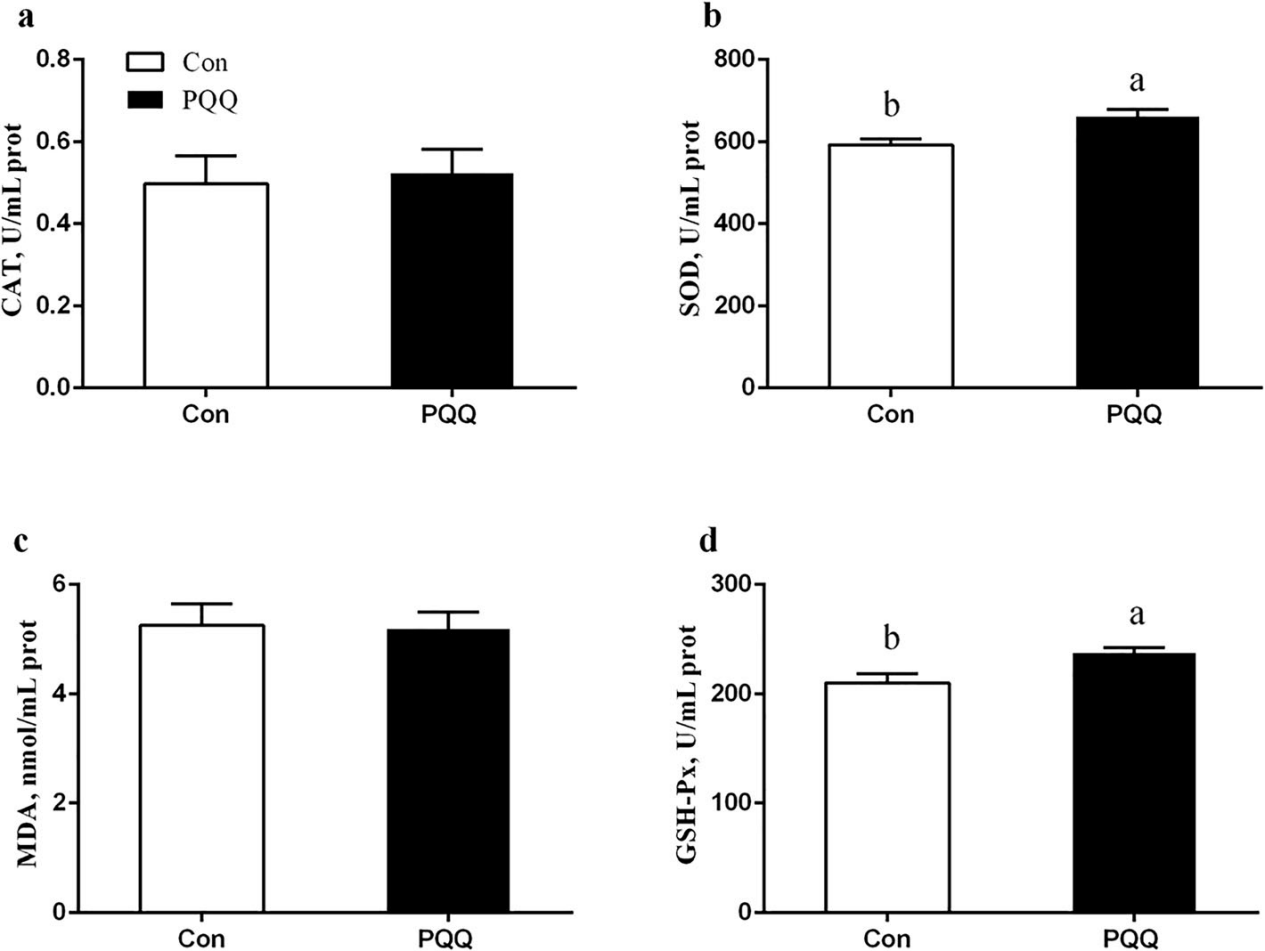
Effects of dietary PQQ·Na_2_ supplementation during gestation and lactation on antioxidant status in milk of sows. Con, control treatment; PQQ, PQQ·Na_2_ treatment; **a** CAT, catalase; **b** SOD, superoxide dismutase; **c** MDA, malondialdehyde; **d** GSH-Px, glutathione peroxidase. All values are expressed as means ± SEM (*n* = 8). a, b Mean values within a column with unlike superscript letters were significantly different (*P* < 0.05)

**Fig. 6:**
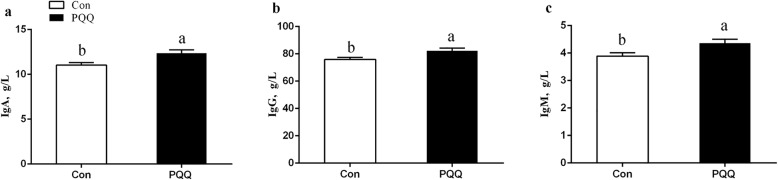
Effects of dietary PQQ·Na_2_ supplementation during gestation and lactation on Immunoglobulin concentrations in the colostrum of the sows. Con, control treatment; PQQ, PQQ·Na_2_ treatment; **a** IgA, immunoglobulin A; **b** IgG, immunoglobulin G; **c** IgM, immunoglobulin M. All values are expressed as means ± SEM (*n* = 8). a, b, c Mean values within a column with unlike superscript letters were significantly different (*P* < 0.05)

Figure 4 shows the protein, lactose, fat and total milk solids content of the sow milk. The concentrations of protein (*P* = 0.011) and total solids (*P* = 0.040) were significantly increased by PQQ·Na_2_ supplementation. The concentrations of fat and lactose were not significant (*P* > 0.05). The SOD (*P* = 0.011) and GSH-Px (*P* = 0.016) activity in milk from the PQQ sows was higher than that in milk from the Con sows, as shown in Fig. 5. The effects of dietary PQQ·Na_2_ supplementation during gestation and lactation on immunoglobulin concentrations in the colostrum of the sows are presented in Fig. 6. The concentrations of IgA, IgG and IgM were significantly (*P* < 0.05) increased in the colostrum with PQQ·Na_2_ supplementation.

### High-throughput transcriptome sequencing and de novo assembly

To understand the molecular basis of the difference in the reproductive performance between Con and PQQ, the placenta was used to build 6 libraries for high-throughput sequencing. We obtained a total of 54.18 Gb of raw data for the 6 samples (Table 4). We discarded low-quality reads, which contained adapters and unknown or low-quality bases, and after stringent quality checks and data cleaning, the clean reads were obtained (Table 4). The GC (guanine + cytosine) contents of these samples were 51.86–53.06%, with an average of 52.51%. The average Q20 and Q3 percentages reached 97.85% and 94.51%, respectively (Table 4).

**Table 4:** Summary of sequencing and assembly data

Item	Sample ID	Clean base	Clean read	GC, %	Q20, %	Q30, %
Con	Con1	8,831,393,714	29,527,850	51.86	97.27	93.57
	Con2	6,702,986,510	22,509,861	53.02	97.91	94.66
	Con3	10,898,101,426	36,518,053	52.23	97.98	94.64
PQQ	PQQ1	9,902,483,500	33,262,116	53.06	98.10	94.92
	PQQ2	7,221,391,084	24,187,833	52.37	97.88	94.59
	PQQ3	7,373,706,268	24,771,902	52.52	97.97	94.73

### Gene annotation and functional classification

**Fig. 7:**
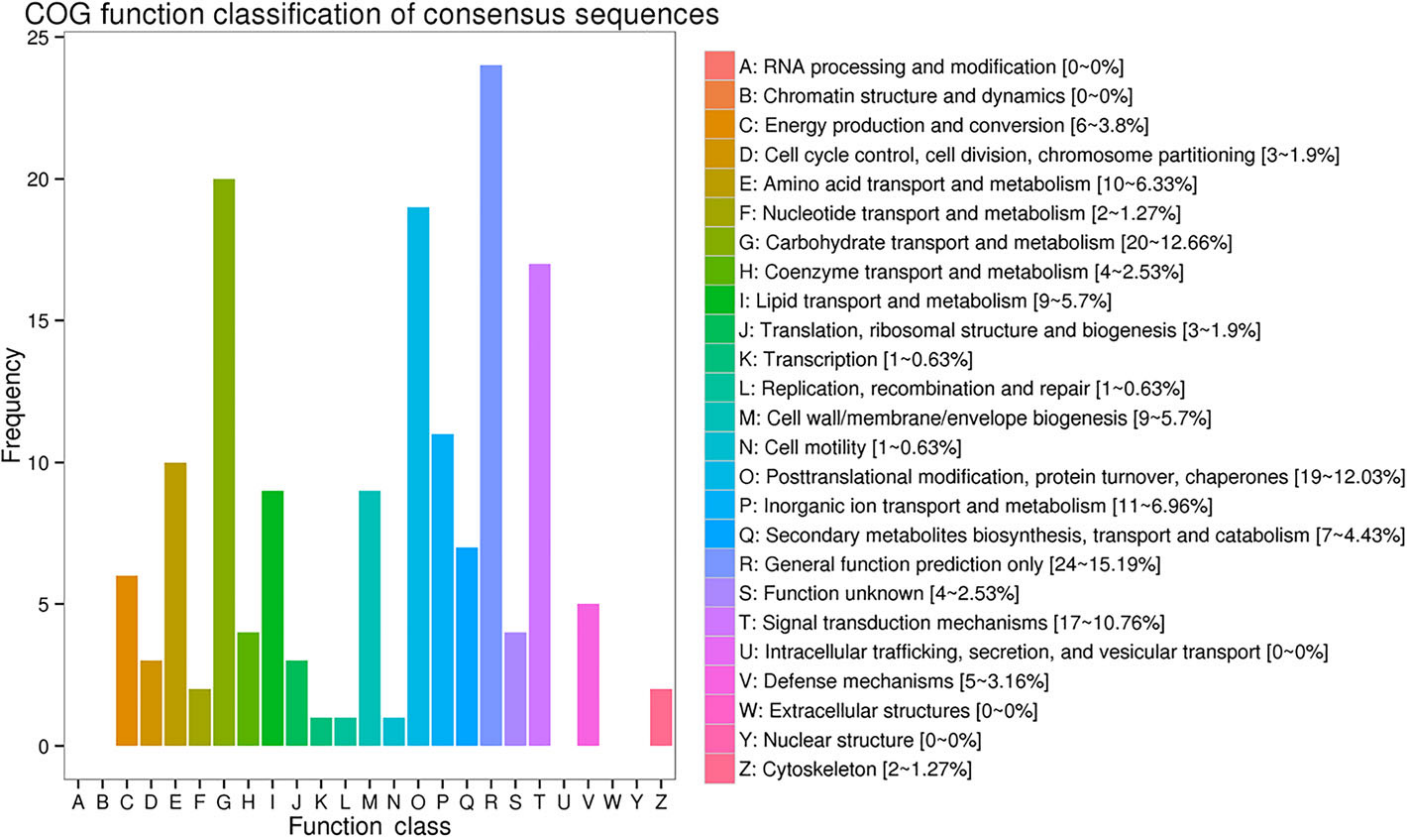
Cluster of orthologous groups (COG) functional classification of unigenes of placenta

**Fig. 8:**
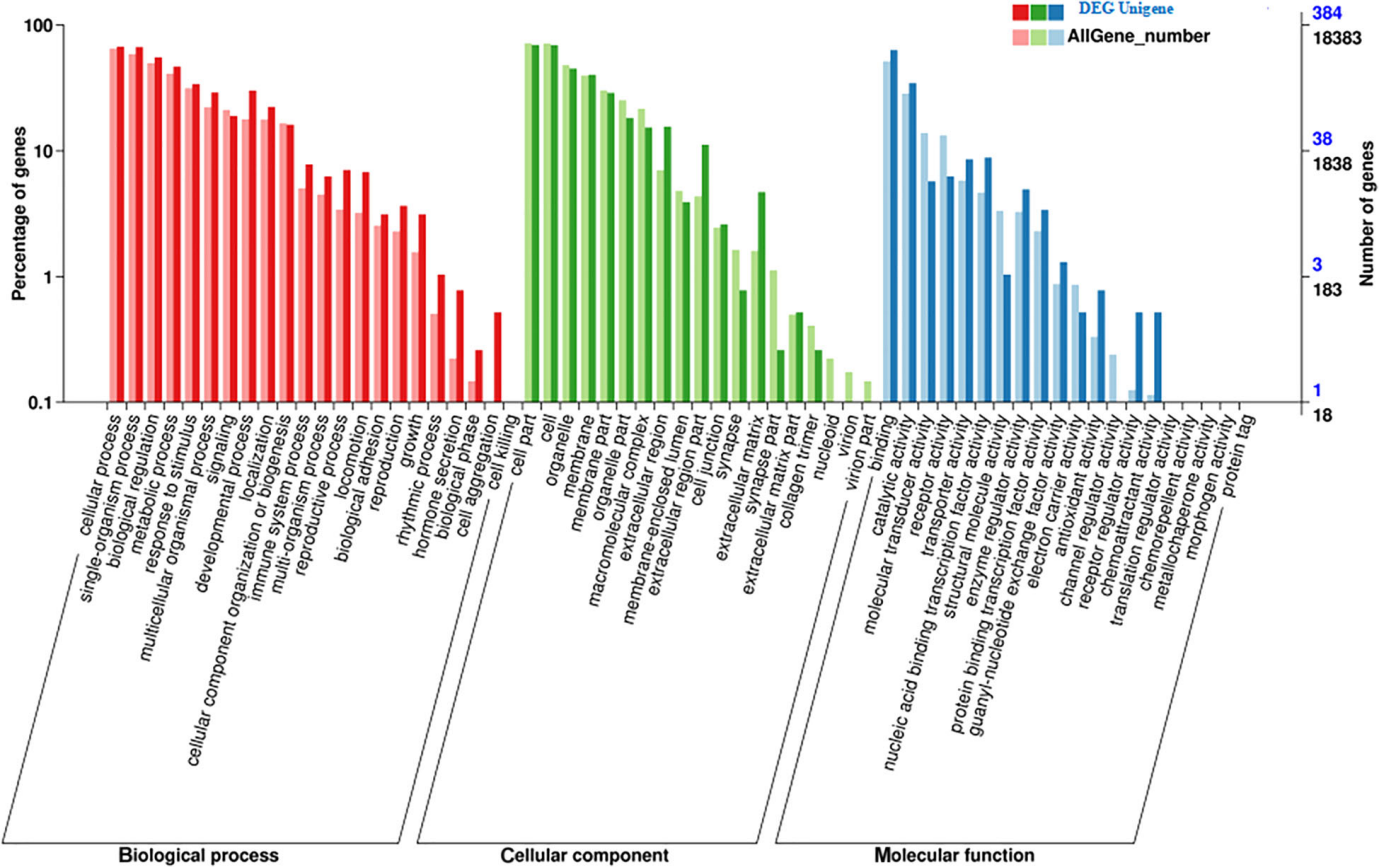
Gene Ontology classification of DEGs between control treatment and PQQ·Na_2_ treatment. Unigenes were annotated in three categories: cellular components, molecular functions, and biological processes. Numbers in black represent the numbers of all unigenes in GO terms, and numbers in blue represent the numers of DEGs in GO terms

All unigenes were aligned to 7 protein databases, including COG, GO, KEGG, KOG, Swiss-Prot, and Nr, using BLASTx with an E-value threshold of 10^− 5^ and Pfam using HMMER with an E-value threshold of 10^− 10^. As shown in Table 5, of 462 unigenes annotated, 448 (96.97%) unigenes had significant BLASTx matches in the Nr database. Based on comparison against the Swiss-Prot database, 370 (80.09%) unigenes had significant matches. In the Pfam and GO databases, 415 (89.93%) and 384 (83.12%) unigenes were also found to have significant matches, respectively, and 316 (68.40%) unigenes were similar to proteins in the KEGG database. To further evaluate the completeness of our transcriptome library and the effectiveness of our annotation process, we searched the annotated sequences for genes with COG (cluster of orthologous groups) classifications, and 145 unigenes were assigned to the COG classification (Fig. 7). Among the 25 COG categories, the cluster for “General function prediction only” (24, 15.19%) represented the largest group, followed by “Carbohydrate transport and metabolism” (20, 12.66%) and “Posttranslational modification, protein turnover, chaperones” (19, 12.03%). Gene ontology (GO) was also used to classify the functions of the predicted unigenes. Based on the sequence homology, 384 sequences were categorized into 61 functional groups (Fig. 6). The assigned functions of the unigenes covered a broad range of GO categories. The unigenes were assigned to three main categories, including the cellular component, molecular function, and biological process categories (Fig. 8). Reproduction (27, 7.03%) and reproduction process (27, 7.03%) were assigned to the biological process category.

**Table 5:** Statistics of annotation analysis of unigenes

Annotated databases	Unigene	Percentage, %
COG	145	31.39
GO	384	83.12
KEGG	316	68.40
KOG	293	63.42
Swiss-Prot	370	80.09
Nr	448	96.97
Pfam	415	89.93
All	462	100

### Analysis of the differentially expressed unigenes (DEGs) by RNA-seq

The gene expression levels of DEGs in the placenta were measured using the calculated values of the RPKM parameter (reads per kilobase of exon per million reads mapped). The TMM (trimmed mean of M-values) method was used to standardize the read counts. Thereafter, the differentially expressed unigene analysis was performed using EB Seq. An absolute value of log_2_ fold change (FC) ≥2 and a false discovery rate (FDR) value of < 0.05 were employed to identify the DEGs and to explore the gene expression levels of DEGs in the placenta between the Con treatment and PQQ treatment. As shown in Additional file 1: Table S1, 462 unigenes were differentially expressed between the Con treatment and PQQ treatment. Among these unigenes, 199 were upregulated, while 263 unigenes were downregulated. By matching DEGs to the KEGG database, we identified 29 pathways (*P <* 0.05, Table 6).

**Table 6:** KEGG pathway enrichment of DEGs

KEGG_pathway	KO_ID	*P*-value
Steroid biosynthesis	ko00100	3.39E-06
Transcriptional misregulation in cancer	ko05202	4.12E-05
Lysosome	ko04142	0.000590824
TGF-beta signaling pathway	ko04350	0.000855626
Bladder cancer	ko05219	0.001822117
Malaria	ko05144	0.00268114
Jak-STAT signaling pathway	ko04630	0.003155387
HIF-1 signaling pathway	ko04066	0.003863436
Signaling pathways regulating pluripotency of stem cells	ko04550	0.005510021
Other glycan degradation	ko00511	0.006945803
Pertussis	ko05133	0.010337763
Insulin signaling pathway	ko04910	0.01140163
FoxO signaling pathway	ko04068	0.013399779
Cytokine-cytokine receptor interaction	ko04060	0.015031804
TNF signaling pathway	ko04668	0.015490522
Terpenoid backbone biosynthesis	ko00900	0.015624507
Renin secretion	ko04924	0.019634552
Salmonella infection	ko05132	0.0219693
Mucin type O-Glycan biosynthesis	ko00512	0.028443139
Hippo signaling pathway	ko04390	0.029186826
Complement and coagulation cascades	ko04610	0.031200574
Amino sugar and nucleotide sugar metabolism	ko00520	0.031819731
African trypanosomiasis	ko05143	0.035721873
ABC transporters	ko02010	0.042072163
Regulation of lipolysis in adipocytes	ko04923	0.044321741
AGE-RAGE signaling pathway in diabetic complications	ko04933	0.046396257
Insulin resistance	ko04931	0.048015681
Folate biosynthesis	ko00790	0.048299174
Chagas disease (American trypanosomiasis)	ko05142	0.049669742

### Validation of differentially expressed genes by qRT-PCR

**Fig. 9:**
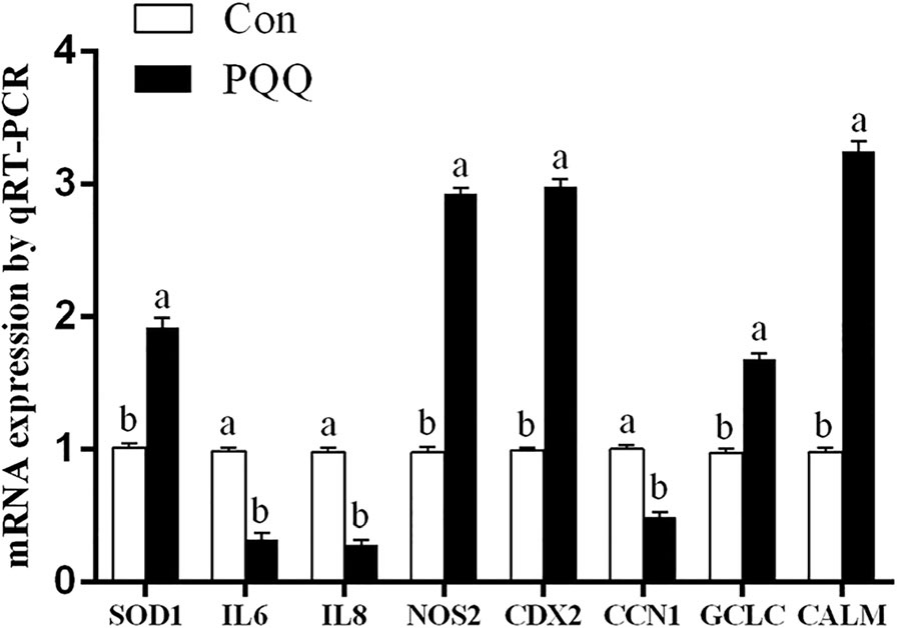
Validation of RNA-seq results with qRT-PCR analysis. The mRNA levels of selected genes were analyzed by qRT-PCR and normalized to GAPDH. IL-6, interleukin 6; IL-8, interleukin 8; SOD1, superoxidedismutase 1; NOS2, nitric oxide synthase 2; CDX2, caudal type homeobox 2; CCN1, cellular communication network factor 1; GCLC, glutamate-cysteine ligase catalytic subunit; CALM, calmodulin. All values are expressed as means ± SEM (*n* = 8). a, b, c Mean values within a column with unlike superscript letters were significantly different (*P* < 0.05)

To validate the results of RNA-seq analysis, qRT-PCR was employed to determine the relative expression of 8 genes in the placenta (Fig. 9), including *SOD1, IL6, IL8, NOS2, CDX2, CCN1, GCLC* and *CALM. IL6, IL8* and *CCN1* were significantly downregulated. *SOD1, NOS2, CDX2, GCLC* and *CALM* were upregulated by PQQ·Na_2_ dietary supplementation (*P* < 0.05), which was consistent with the data from the RNA-seq analysis. Differences in the magnitude of fold-change values were likely due to differences in detection sensitivity of the two methods. In addition, correlation analysis demonstrated that the values of log_2_ (fold-change) obtained from RNA-seq and qRT-PCR were significantly correlated (*R*^2^ = 0.94). Thus, our RNA-seq analysis results are valid.

## Discussion

The use of PQQ in nutrition is increasingly being discussed in the literature [19, 20]. Jonscher KR et al. [21] reported that PQQ treatment led to a significant increase in placental weight and placental surface area. Yin et al. [22] suggested that the dietary supplementation of 1.50 mg/kg PQQ·Na_2_ is the lowest functional dose to improve the growth performance for weaned pigs and the expression of the jejunal tight junction protein *ZO-1* was significantly higher in pigs with PQQ·Na_2_ supplementation. Zhang et al. [23] suggested that dietary 1.6 mg/kg PQQ·Na_2_ supplementation during gestation and lactation of female rats can significantly increase the number of viable foetuses per litter, born alive litter weight and the mRNA expression levels of *CAT, GPX2* and *SOD1* in the placenta. Steinberg et al. [24] observed that 0.4 mg PQQ/kg in an amino acid-based diet optimized reproduction. To our knowledge, this is the first study to examine the effects of dietary PQQ·Na_2_ supplementation during gestation and lactation in sows. Several reports have investigated DEGs from reproductive tissues, such as the endometrium, in pigs [25]. DEGs in the placenta have rarely been demonstrated using RNA-seq.

During pregnancy and lactation in sows, high energy and oxygen levels are required to satisfy increasing metabolic burdens for fetal growth, placenta development and milk production, which could cause elevated ROS production [26] Oxidative stress results from increased production of reactive oxygen species (ROS) or a decrease in antioxidant defense. Oxidative damage is a strong indicator of the health status and wellbeing of animals [27]. A recent study showed that pregnant sows had elevated oxidative stress during late gestation and lactation, which was responsible for impaired milk production, reproductive performance, and longevity of sows [28]. The protective role of an antioxidant against oxidative stress in sows has been clearly demonstrated [29], and dietary addition of antioxidants can reduce oxidative stress and improve the reproductive performance of sows [25]. Thus, dietary antioxidant concentrations need to be added in sow diets, especially to prevent excessive oxidative stress during gestation and lactation. In this study, we found that the antioxidant status of sows, including in the plasma, placenta and milk, was partially improved and oxidative stress markers were partially reduced by dietary PQQ·Na_2_ supplementation. SOD is known to serve a protective function for the elimination of reactive free radicals and, therefore, it represents an important antioxidant defense in nearly all cells exposed to oxygen. GSH forms an important part of the nonenzymatic antioxidants [30]. Similar to other sulfhydryl-containing products, GSH also has regulatory and protective roles in the body, as it establishes the defenses of the body against tissue injury due to chemicals through its ROS scavenging, cell viability and membrane-stabilizing effects [31]. In this study, dietary PQQ·Na_2_ supplementation can increase the antioxidant enzyme activities of SOD and GSH-Px in the placenta, plasma and milk. A previous study has demonstrated that SOD activity can be upregulated by PQQ·Na_2_ treatment [32], and this is consistent with our study results. The antioxidant enzymes SOD and CAT are considered the first line of defense against ROS. In this study, CAT was increased in the plasma (90 d of gestation). MDA is an end product of free-radical chain reactions and lipid peroxidation [33], so it is frequently used in the measurement of lipid peroxide levels, and it correlates well with the degree of lipid peroxidation [34]. In the present study, the MDA levels in the placenta and plasma (21 d of lactation) was significantly decreased by PQQ·Na_2_ supplementation. PQQ acts as an antioxidant by scavenging O_2_^−^ and protects mitochondria from oxidative stress-induced damage. In our study, the total piglets born, number of piglets born alive and born alive litter weight were significantly increased. Evidence has shown that supplementation with antioxidants, such as selenium, vitamin E and vitamin C, improves antioxidant status and reproductive performance in sows [35, 36]. Similarly, a growing number of studies have demonstrated that certain functional substances, such as chitosan [37], resveratrol [38], and isoflavone [25], alleviate oxidative stress and improve the reproductive performance of sows. Our results are in alignment with these studies and demonstrate that dietary PQQ·Na_2_ exerts a beneficial role in antioxidant defense and the reproductive performance of sows.

Colostrum is of great importance for the growth and development of piglets during and after lactation [39]. Because neither creep feed nor milk replacer was used for the suckling piglets in this study, the sow milk served asthe sole source of nutrients and antioxidants for the piglets. Therefore, litter performance reflected the nutrient composition and antioxidant status of the colostrum and sow milk only. There is evidence that sow’s colostrum and milk include various antioxidants, including SOD, GSH-Px and GSH [25, 40]. In the present study, SOD and GSH-Px activity in the colostrum was significantly increased by dietary PQQ·Na_2_ during gestation and lactation. Milk antioxidants, which provide antioxidant protection to suckling piglets, may be as important as nutrients or immunological factors in protecting the health of the neonatal piglet [35]. Colostrum is the main external resource providing piglets with nutrients and maternal immune molecules. Newborn piglets can hardly obtain passive immunity from the maternal blood during the fetal period because of the special epitheliochorial structure of the pig placenta. Before their own immune system is fully developed, colostrum is the sole external resource which provides piglets with nutrients and maternal immune molecules [41]. In the present study, the concentrations of protein and total solids were increased. The high total solids and protein content in colostrum is mostly due to immunoglobulin [42]. Immunoglobulin in colostrum, mainly IgG, provides humoral immune protection for the newborn piglet until its own immune system has sufficiently matured to respond to antigens [43]. Additionally, immunoglobulin in the colostrum and milk could increase susceptibility to infection in newborn animals, not only in the immediate postnatal period but also after weaning [44]. IgA is the main immunoglobulin of milk and could protect piglets against local pathogens, commensal bacteria and food antigens in the digestive tract [45]. In our study, the concentrations of IgG, IgA and IgM in the colostrum were significantly increased by PQQ·Na_2_ supplementation, which might aid the humoral and mucosal immunity of piglets.

Nitric oxide (NO) is a pleiotropic regulator and is pivotal to numerous biological processes, including vasodilation, neurotransmission, and macrophage-mediated immunity [46]. NO plays an important role in regulating placental-fetal blood flow, contributing to maternal systemic vasodilatation during pregnancy and regulating uterine and fetal placental blood flow [47]. It has been reported that antioxidant activity inhibits lipid peroxidation, increases nitric oxide (NO) production, reduces oxidation of low-density lipoproteins, and preserves superoxide dismutase (SOD) activity [48]. In the present study, PQQ increased antioxidants and iNOS in the placenta and plasma, which can increase NO. The increased NO plays an important part in improving placental vascular function and promoting the nutrient supply to the fetus. NO plays an important role in vasodilatation and regulates uterine blood flow, promoting the transfer of nutrients to the fetus [49]. It has reported that NO is a key regulator of angiogenesis and embryogenesis as well as placental and fetal growth [50]. In addition, NO might positively influence the final litter size, as it also induces follicle rupture *in vitro* in rabbit ovaries [51].

PQQ has attracted considerable attention, as it is important for mammalian growth, development, reproduction and immune function. However, the mechanism by which PQQ is beneficial to reproduction is not clear. Thus, we performed an RNA-seq analysis on the placenta of sows. We found 462 DEGs, including 199 upregulated genes and 263 downregulated genes. To validate the DEGs identified by RNA-seq analysis, we confirmed the expression levels of 8 genes by qRT-PCR. Comparison of the results obtained using the two methods revealed similar trends, confirming their validity.

The placenta is an important endocrine organ throughout pregnancy and markedly affects fetal health by supplying nutrients. Among the 25 COG categories, we found that the cluster for “General function prediction only” (24, 15.19%) represented the largest group, followed by “Carbohydrate transport and metabolism” (20, 12.66%). Effects of inadequate dietary protein and carbohydrate intake on maternal venous plasma amino acid profiles occurred from early pregnancy on, probably with impacts on placental growth and function and eventually on fetal development [52]. By matching DEGs to the KEGG database, we identified 29 pathways. From these pathways, the “Steroid biosynthesis”, “TGF-beta signaling pathway”, “Jak-STAT signaling pathway”, “Insulin signaling pathway”, “Hippo signaling pathway” and “Folate biosynthesis” are closely related to reproductive performance. Ovarian and placental steroids are essential for the maintenance of pregnancy. In some mammals it is evident that the placenta is responsible for the production of steroids [53]. The TGF-beta superfamily has been observed to oppose trophoblastic migration, suggesting an intricate balance of growth factor expression that is fundamental for placental health and embryo development [54]. Tojo reported that the entire TGF-β signal transduction pathway is essential to proper development and embryonic survival [55]. The JAK-STAT pathway is frequently activated and is indispensable and pivotal in many biological processes, including immunity and the inflammatory response [56]. In previous studies we showed that PQQ regulates intracellular JAK/STAT signaling pathway activation. Eskouhie Tchaparian et al. reported changes in gene expression patterns and transcriptional networks that respond to dietary PQQ restriction or pharmacological administration, they found JAK/STAT pathways seem particularly influenced by PQQ [57]. Zhang reported that the insulin signaling pathway was down-regulated in the placenta of women with gestational diabetes mellitus [58]. Dysregulation of Hippo signaling component genes can result in embryonic lethality [59]. Folates are needed for fetal growth and placental development, since they activate cell growth and biosynthetic processes that are essential during pregnancy [60].

GO analyses showed that 27 DEGs were involved in reproduction. Among these DEGs, the ovo-like zinc finger 2 (*OVOL2*), distal-less homeobox 1 (*DLX1*), distal-less homeobox 2 (*DLX2*), distal-less homeobox 5 (*DLX5*), msh homeobox 2 (*MSX2*), caudal type homeobox 2 (*CDX2*), nitric oxide synthase 2 (*NOS2*) and calmodulin (*CALM*) were upregulated. Unezaki reported that *ovol2* function is required for endothelial cell growth during heart development and angiogenesis of extraembryonic and embryonic vessels [61]. The Dlx genes encode a family of transcription factors with important roles in patterning and differentiation during vertebrate embryogenesis [62]. Previous studies indicate that *MSX2* plays an important role in mammalian embryonic diapause [63, 64]. Sakurai suggested that *CDX2* is essential for early development and gene expression and is involved in differentiation of the inner cell mass and trophectoderm lineages in embryos [65]. Kwon showed, by comparing sperm proteins from different litter sizes, that *CALM* was highly expressed in high swine litter sizes and was positively related to litter size [66]. Furthermore, antioxidant genes, such as *SOD1, GCLC* and *DHCR24*, were also upregulated by PQQ·Na_2_ supplementation. In sows, supplementation improves antioxidant levels and alleviates oxidative stress effectively, which are beneficial to litter size and piglet growth. *DHCR24* exerts cytoprotective effects against endoplasmic reticulum stress by eliminating ROS. *DHCR24* can scavenge hydrogen peroxide (H_2_O_2_), protecting cells from oxidative stress-induced apoptosis [67]. *GCLC* is an important part of GSH, which is an extremely important antioxidant. It not only scavenges free radicals but also maintains the redox-sensitive active sites of many enzymes from an oxidized form to a reduced form [68]. The supply of glucose to the embryo from the maternal circulation is important for normal metabolism and growth, as glucose constitutes the main energy substrate during embryogenesis [69]. The solute carrier (SLC) family (including *SLC1A1, SLC7A4, SLC7A10* and *SLC19A1*) was upregulated, and many nutrient carriers and growth factors decrease as the dam is exposed to stress. Oxidative stress, defined as an imbalance between the production of free radicals and reactive metabolites, is closely related to inflammation. GO analysis identified genes involved in the inflammatory response (including *IL6*, *IL8*, *IL11* and *CCN1*) that were downregulated. KEEG pathway analysis showed that the cytokine-cytokine receptor interaction pathway was significantly reduced, which indicated that the inflammatory state of the placenta was alleviated by maternal PQQ·Na_2_ supplementation. A previous study showed that PQQ can reduce the expression of inflammatory cytokine genes [20] which was consistent with our study.

## Conclusions

In conclusion, our results have shown that dietary 20 mg/kg PQQ·Na_2_ supplementation during gestation and lactation in sows can significantly increase the total piglets born, number of piglets born alive and born alive litter weight. It also increased antioxidant levels in the placenta, plasma and milk. The concentration of NO was significantly increased in the plasma and placenta. RNA-seq analysis showed that 462 unigenes were differentially expressed between Con treatment and PQQ treatment. Among these unigenes, 199 were upregulated, while 263 unigenes were downregulated. By matching DEGs to the KEGG database, we identified 29 pathways. These provide a theoretical basis to further explore the effect of PQQ on the reproductive performance mechanism of sows. The present study can provide a scientific basis for dietary PQQ·Na_2_ supplementation in sows.

## Additional file

Additional file 1: **Table S1.** Analysis of the differentially expressed unigenes (DEGs) by RNA-seq. (DOC 644 kb)

## Data Availability

The datasets produced and/or analyzed during the current study are available from the corresponding author on reasonable request.

## Abbreviations

ANOVA: Analysis of variance
CALM: Calmodulin
CAT: Catalase
CCN1: Cellular communication network factor 1
CDX2: Caudal type homeobox 2
CDX2: Caudal type homeobox 2
COG: Clusters of Orthologous Groups
Con: Contral
DEGs: Differentially expressed genes
DHCR24: 24-dehydrocholesterol reductase
DLX1: Distal-less homeobox 1
DLX2: Distal-less homeobox 2
DLX5: Distal-less homeobox 5
FC: Fold change
FDR: False discovery rate
FPKM: Fragments per kilobase of transcript per million fragments mapped
GCLC: Glutamate-cysteine ligase catalytic subunit
GO: Gene Ontology
GSH-Px: Glutathione peroxidase
IgA: Immunoglobulin A
IgG: Immunoglobulin G
IgM: Immunoglobulin M
IL-11: Interleukin 11
IL-6: Interleukin 6
IL-8: Interleukin 8
iNOS: Inducible NOS
KEGG: Kyoto Encyclopedia of Genes and Genomes
MDA: Malondialdehyde
MSX2: msh homeobox 2
NCBI: National Center for Biotechnology Information
NO: Nitric oxide
NOS2: Nitric oxide synthase 2
Nr: Non-redundant protein
OVOL2: Ovo like zinc finger 2
PQQ: Pyrroloquinoline quinone
PQQ·Na_2_: Pyrroloquinoline quinone disodium
qRT-PCR: Quantitative real-time PCR
RNA-seq: RNA-seq
ROS: Reactive oxygen species
SEM: Standard error of the mean
SLC19A1: Solute carrier family 19 member 1
SLC1A1: Solute carrier family 1 member 1
SLC7A10: Solute carrier family 7 member 10
SLC7A4: Solute carrier family 7 member 4
SOD: Superoxide dismutase
SOD1: Superoxidedismutase 1

## References

[1] Zhang Shuna, Yang Jiahao, Wang Lei, Li Zhenzhu, Pang Panfei, Li Fenge (2018). SLA-11 mutations are associated with litter size traits in Large White and Chinese DIV pigs. Animal Biotechnology.

[2] Balcells I, Castelló A, Mercadé A (2011). Analysis of porcine MUC4 gene as a candidate gene for prolificacy QTL on SSC13 in an Iberian × Meishan F2 ovulation. BMC Genetics.

[3] Huang L., Yin Z. J., Feng Y. F., Zhang X. D., Wu T., Ding Y. Y., Ye P. F., Fu K., Zhang M. Q. (2016). Identification and differential expression of microRNAs in the ovaries of pigs (Sus scrofa) with high and low litter sizes. Animal Genetics.

[4] Ren P., Yang X.J., Railton R., Jendza J., Anil L., Baidoo S.K. (2018). Effects of different levels of feed intake during four short periods of gestation and housing systems on sows and litter performance. Animal Reproduction Science.

[5] Leddy MA, Power ML, Schulkin J (2008). The impact of maternal obesity on maternal and fetal health. Rev Obstet Gynecol.

[6] Tarrade A, Panchenko P, Junien C (2015). Placental contribution to nutritional programming of health and diseases: epigenetics and sexual dimorphism. J Exp Biol.

[7] Lee Dong-Gi, Nam Juhyun, Kim Sam Woong, Kang Young-Moon, An Hyun Joo, Kim Chul Wook, Choi Jong-Soon (2015). Proteomic analysis of reproduction proteins involved in litter size from porcine placenta. Bioscience, Biotechnology, and Biochemistry.

[8] Zhang Y, Feustel PJ, Kimelberg HK (2006). Neuroprotection by pyrroloquinoline quinone (PQQ) in reversible middle cerebral artery occlusion in the adult rat. Brain Res.

[9] Killgore J, Smidt C, Duich L (1989). Nutritional importance of pyrroloquinoline quinone. Science.

[10] Akagawa Mitsugu, Nakano Masahiko, Ikemoto Kazuto (2015). Recent progress in studies on the health benefits of pyrroloquinoline quinone. Bioscience, Biotechnology, and Biochemistry.

[11] Ikemoto K, Mori S, Mukai K (2017). Synthesis and crystal structure of pyrroloquinoline quinol (PQQH2) and pyrroloquinoline quinone (PQQ). Acta Crystallogr Sect B: Struct Sci.

[12] Hwang P, Willoughby DS. Mechanisms behind Pyrroloquinoline Quinone supplementation on skeletal muscle mitochondrial biogenesis: possible synergistic effects with exercise. J Am Coll Nutr. 2018:1–11. 10.1080/07315724.2018.1461146 29714638

[13] Mitchell AE, Jones AD, Mercer RS (1999). Characterization of Pyrroloquinoline Quinone amino acid derivatives by electrospray ionization mass spectrometry and detection in human Milk. Anal Biochem.

[14] Samuel KG, Zhang HJ, Wang J (2014). Effects of dietary pyrroloquinoline quinone disodium on growth performance, carcass yield and antioxidant status of broiler chicks. Animal.

[15] O’Loughlin A, Lynn DJ, Mark MG (2012). Transcriptomic analysis of the stress response to weaning at housing in bovine leukocytes using RNA-seq technology. BMC Genomics.

[16] Gao W, Sun HX, Xiao H (2014). Combining metabolomics and transcriptomics to characterize tanshinone biosynthesis inSalvia miltiorrhiza. BMC Genomics.

[17] Noji N, Nakamura T, Kitahata N (2007). Simple and sensitive method for Pyrroloquinoline Quinone (PQQ) analysis in various foods using liquid chromatography/electrospray-ionization tandem mass spectrometry. J Agric Food Chem.

[18] Grabherr MG, Haas BJ, Yassour M (2011). Full-length transcriptome assembly from RNA-Seq data without a reference genome. Nat Biotechnol.

[19] Wang J, Zhang HJ, Xu L (2016). Dietary supplementation of pyrroloquinoline quinone disodium protects against oxidative stress and liver damage in laying hens fed an oxidized sunflower oil-added diet. Animal.

[20] Harris CB, Chowanadisai W, Mishchuk DO (2013). Dietary pyrroloquinoline quinone (PQQ) alters indicators of inflammation and mitochondrial-related metabolism in human subjects[J]. J Nutr Biochem.

[21] Jonscher KR, Stewart MS, Alfonso-Garcia A (2017). Early PQQ supplementation has persistent long-term protective effects on developmental programming of hepatic lipotoxicity and inflammation in obese mice. FASEB J.

[22] Yin Xindi, Ming Dongxu, Bai Lili, Wu Fei, Liu Hu, Chen Yifan, Sun Linlin, Wan Yidong, Thacker Philip Alfred, Wu Guoyao, Wang Fenglai (2018). Effects of pyrroloquinoline quinone supplementation on growth performance and small intestine characteristics in weaned pigs1,2. Journal of Animal Science.

[23] Zhang Boru, Yang Wei, Zhang Hongyun, He Shiqi, Meng Qingwei, Chen Zhihui, Shan Anshan (2019). Effect of pyrroloquinoline quinone disodium in female rats during gestating and lactating on reproductive performance and the intestinal barrier functions in the progeny. British Journal of Nutrition.

[24] Steinberg FM (1994). Dietary pyrroloquinoline quinone : growhth and immune response in BALB/c mice. J Nutr.

[25] Chen X, Li A, Chen W, et al. Differential gene expression in uterine endometrium during implantation in pigs. Biol Reprod. 2015;92(2):52–2. 10.1095/biolreprod.114.123075 25519183

[26] Hu YJ, Gao KG, Zheng CT (2015). Effect of dietary supplementation with glycitein during late pregnancy and lactation on antioxidative indices and performance of primiparous sows. J Anim Sci.

[27] Agarwal A, Makker K, Sharma R (2010). Clinical relevance of oxidative stress in male factor infertility: an update. Am J Reprod Immunol.

[28] Lapointe J (2014). Mitochondria as promising targets for nutritional interventions aiming to improve performance and longevity of sows. J Anim Physiol Anim Nutr.

[29] Agarwal A, Aponte-Mellado A, Premkumar BJ (2012). The effects of oxidative stress on female reproduction: a review. Reprod Biol Endocrinol.

[30] Cheung CCC, Zheng GJ, Li AMY (2001). Relationships between tissue concentrations of polycyclic aromatic hydrocarbons and antioxidative responses of marine mussels, Perna viridis. Aquatic Toxicology (Amsterdam).

[31] Szabó S (1984). Role of sulfhydryls and early vascular lesion in gastric mucosal injury. Acta Physiol Hung.

[32] Zhang Q, Ding M, Gao XR (2012). Pyrroloquinoline quinone rescues hippocampal neurons from glutamate-induced cell death through activation of Nrf2 and up-regulation of antioxidant genes. Genet Mol Res.

[33] Tomita M, Okuyama T, Kawai S (1990). Determination of malonaldehyde in oxidized biological materials by high-performance liquid chromatography. J Chromatogr A.

[34] Wei B, Nie S, Meng Q (2016). Effects of l-carnitine and/or maize distillers dried grains with solubles in diets of gestating and lactating sows on the intestinal barrier functions of their offspring. Br J Nutr.

[35] Chen J, Han JH, Guan WT (2016). Selenium and vitamin E in sow diets: I. effect on antioxidant status and reproductive performance in multiparous sows. Anim Feed Sci Tech.

[36] Pinellisaavedra A (2003). Vitamin E in immunity and reproductive performance in pigs. Reprod Nutr Dev.

[37] Xie C, Wu X, Long C (2016). Chitosan oligosaccharide affects antioxidant defense capacity and placental amino acids transport of sows. BMC Vet Res.

[38] Qingwei, Meng, Gaoqiang, et al. Dietary resveratrol improves antioxidant status of sows and piglets and regulates antioxidant gene expression in placenta by Keap1-Nrf2 pathway and Sirt1. Journal of livestock and biotechnology, 2018(3):639–651. 10.1186/s40104-018-0248-y PMC5909222 29713468

[39] Hurley Walter L., Theil Peter K. (2011). Perspectives on Immunoglobulins in Colostrum and Milk. Nutrients.

[40] Lipkoprzybylska J, Kankofer M. Antioxidant defence of colostrum and milk in consecutive lactations in sows[J]. Ir Vet J. 2012;65(1):4-4. 10.1186/2046-0481-65-4 PMC3338395 22429994

[41] Tan L, Wei T, Yuan A (2017). Dietary supplementation of Astragalus polysaccharides enhanced immune components and growth factors EGF and IGF-1 in sow colostrum. J Immunol Res.

[42] Wang LS, Su BC, Shi Z (2013). Dietary supplementation with maize distillers dried grains with solubles during late gestation and lactation: effects on sow and litter performance, and on colostrum and milk composition. Anim Feed Sci Technol.

[43] Liu ST, Hou WX, Cheng SY (2014). Effects of dietary citric acid on performance, digestibility of calcium and phosphorus, milk composition and immunoglobulin in sows during late gestation and lactation. Anim Feed Sci Technol.

[44] Varley MA, Towle AMA (2010). Artificial rearing of piglets: the administration of two sources of immunoglobulins after birth. Anim Prod.

[45] Brandtzaeg P (2010). The Mucosal Immune System and Its Integration with the Mammary Glands. J Pediatr.

[46] Rao Chinthalapally V. (2004). Nitric oxide signaling in colon cancer chemoprevention. Mutation Research/Fundamental and Molecular Mechanisms of Mutagenesis.

[47] Sladek SM, Magness RR, Conrad KP (1997). Nitric oxide and pregnancy. Am J Phys.

[48] Mason RP (2002). Mechanisms of plaque stabilization for the dihydropyridine calcium channel blocker amlodipine: review of the evidence. Atherosclerosis.

[49] Moncada S, Higgs EA (1995). Molecular mechanisms and therapeutic strategies related to nitric-oxide. FASEB J.

[50] Wu G, Bazer FW, Cudd TA (1980). Maternal nutrition and fetal development. Early Hum Dev.

[51] Yamauchi J. (1997). Effects of Nitric Oxide on Ovulation and Ovarian Steroidogenesis and Prostaglandin Production in the Rabbit. Endocrinology.

[52] Strakovsky RS, Zhou D, Pan YX (2010). A low-protein diet during gestation in rats activates the placental mammalian amino acid response pathway and programs the growth capacity of offspring. J Nutr.

[53] Braun BC, Zschockelt L, Dehnhard M (2012). Progesterone and estradiol in cat placenta--biosynthesis and tissue concentration. J Steroid Biochem Mol Biol.

[54] Nikuei P, Malekzadeh K, Rajaei M (2015). The imbalance in expression of angiogenic and anti-angiogenic factors as candidate predictive biomarker in preeclampsia.

[55] Tojo M, Takebe A, Takahashi S (2012). Smad7-deficient mice show growth retardation with reduced viability. J Biochem.

[56] O’Shea JJ, Schwartz DM, Villarino AV (2015). The JAK-STAT pathway: impact on human disease and therapeutic intervention*. Annu Rev Med.

[57] Tchaparian E, Marshal L, Cutler G (2010). Identification of transcriptional networks responding to pyrroloquinoline quinone dietary supplementation and their influence on thioredoxin expression, and the JAK/STAT and MAPK pathways. Biochem J.

[58] Zhang Wanyi, Su Rina, Lin Li, Yang Huixia (2018). ARHGEF11 affecting the placental insulin signaling pathway in fetal macrosomia of normal glucose tolerance pregnant women. Placenta.

[59] Halder G, Johnson RL (2011). Hippo signaling: growth control and beyond. Development.

[60] Castaño E, Caviedes L, Hirsch S (2017). Folate Transporters in Placentas from Preterm Newborns and Their Relation to Cord Blood Folate and Vitamin B12 Levels. Plos One.

[61] Unezaki S, Horai R, Sudo K, Iwakura Y, Ito S (2007). Ovol2/Movo, a homologue of Drosophila ovo, is required for angiogenesis, heart formation and placental development in mice. Genes Cells.

[62] Coubrough Melissa L., Bendall Andrew J. (2006). Impaired nuclear import of mammalian Dlx4 proteins as a consequence of rapid sequence divergence. Experimental Cell Research.

[63] Cha J, Sun X, Bartos A (2013). A new role for muscle segment homeobox genes in mammalian embryonic diapause. Open Biol.

[64] Renfree MB, Shaw G (2014). Embryo-endometrial interactions during early development after embryonic diapause in the marsupial tammar wallaby. Int J Dev Biol.

[65] Sakurai N , Takahashi K , Emura N , et al. The Necessity of OCT-4 and CDX2 for Early Development and Gene Expression Involved in Differentiation of Inner Cell Mass and Trophectoderm Lineages in Bovine Embryos. Cellular Reprogramming, 2016:cell.2015.0081. 10.1089/cell.2015.0081 27500421

[66] Kwon WS, Rahman MS, Lee JS (2015). Discovery of predictive biomarkers for litter size in boar spermatozoa. Mol Cell Proteomics.

[67] Lu X, Kambe F, Cao X (2008). 3beta-Hydroxysteroid-delta24 reductase is a hydrogen peroxide scavenger, protecting cells from oxidative stress-induced apoptosis. Endocrinology.

[68] Jian Z, Mei P, Ki K (2014). Fucoxanthin enhances the level of reduced glutathione via the Nrf2-mediated pathway in human keratinocytes. Marine Drugs.

[69] Jensen Vivi F.H., Mølck Anne-Marie, Lykkesfeldt Jens, Bøgh Ingrid B. (2018). Effect of maternal hypoglycaemia during gestation on materno-foetal nutrient transfer and embryo-foetal development: Evidence from experimental studies focused primarily on the rat. Reproductive Toxicology.

