# Supplementary material for: Transcriptome analysis of the effect of pyrroloquinoline quinone disodium (PQQ·Na2) on reproductive performance in sows during gestation and lactation
Source: J Anim Sci Biotechnol. 2019 Aug 7;10:62. doi: 10.1186/s40104-019-0369-y (PMC6685232; doi:10.1186/s40104-019-0369-y)
Supplement: Supplementary file 1 — Table S1. Analysis of the differentially expressed unigenes (DEGs) by RNA-seq. (DOC 644 kb) [file 40104_2019_369_MOESM1_ESM.doc]

**Supplementary Table S1. Analysis of the differentially expressed unigenes (DEGs) by RNA-Seq**

| #ID | gene_name | log2FC | regulated |
| --- | --- | --- | --- |
| ENSSSCG00000023907 | AFAP1 | -1.287453442 | down |
| ENSSSCG00000010530 | CRTAC1 | -2.407283128 | down |
| ENSSSCG00000017728 | MYO1D | -1.794299037 | down |
| ENSSSCG00000028277 | ENSSSCG00000028277 | 1.794009903 | up |
| ENSSSCG00000013775 | ADGRE5 | -1.170846797 | down |
| ENSSSCG00000013888 | JAK3 | -1.666213812 | down |
| ENSSSCG00000012679 | GPC4 | -1.668047286 | down |
| ENSSSCG00000001235 | TRIM15 | 1.362305579 | up |
| ENSSSCG00000038732 | AGAP2 | 2.155011189 | up |
| ENSSSCG00000015953 | DLX1 | 1.747083808 | up |
| ENSSSCG00000016443 | TMEM176A | -1.713680987 | down |
| ENSSSCG00000031970 | RASSF5 | -1.299156001 | down |
| ENSSSCG00000005484 | ZNF618 | -1.336757835 | down |
| Sus_scrofa_newGene_33705 | -- | 1.221860257 | up |
| ENSSSCG00000017778 | DHRS13 | 1.107448969 | up |
| ENSSSCG00000013566 | INSR | -1.515460227 | down |
| Sus_scrofa_newGene_41138 | -- | -1.853185143 | down |
| ENSSSCG00000028741 | ENSSSCG00000028741 | 1.491065371 | up |
| ENSSSCG00000038948 | ETS2 | -1.346960514 | down |
| ENSSSCG00000016284 | ENSSSCG00000016284 | 1.518214914 | up |
| ENSSSCG00000005688 | PTGES | 1.048739335 | up |
| Sus_scrofa_newGene_41946 | -- | -1.506033313 | down |
| ENSSSCG00000012137 | BMX | -1.793662503 | down |
| ENSSSCG00000006455 | ENSSSCG00000006455 | -1.741097263 | down |
| Sus_scrofa_newGene_19491 | -- | -2.600442753 | down |
| ENSSSCG00000013388 | PDE3B | -1.112609478 | down |
| Sus_scrofa_newGene_55579 | -- | 1.716374115 | up |
| ENSSSCG00000010728 | CPXM2 | -2.073595372 | down |
| ENSSSCG00000024736 | ACP4 | 1.500595681 | up |
| Sus_scrofa_newGene_53885 | -- | -2.484969527 | down |
| ENSSSCG00000003888 | STIL | -1.067693042 | down |
| ENSSSCG00000009523 | GGACT | 1.505516372 | up |
| ENSSSCG00000007850 | ANKS4B | 2.147472012 | up |
| ENSSSCG00000009864 | MED13L | -1.119139454 | down |
| ENSSSCG00000012915 | CLCF1 | -1.524151898 | down |
| ENSSSCG00000001917 | CD276 | -1.505587136 | down |
| ENSSSCG00000009699 | HPGD | -1.43141929 | down |
| ENSSSCG00000040186 | C12orf57 | 2.366702102 | up |
| ENSSSCG00000016868 | ANXA2R | -2.00559979 | down |
| ENSSSCG00000013004 | TM7SF2 | 1.64189629 | up |
| ENSSSCG00000028512 | LDLR | -1.672951787 | down |
| ENSSSCG00000006535 | PMVK | 1.003805687 | up |
| ENSSSCG00000026996 | ABCC4 | -1.852493129 | down |
| ENSSSCG00000015747 | ENSSSCG00000015747 | 1.742313152 | up |
| ENSSSCG00000002864 | SLC7A10 | 1.582332648 | up |
| ENSSSCG00000014727 | ENSSSCG00000014727 | -1.644176813 | down |
| ENSSSCG00000008867 | CTSO | -1.280595381 | down |
| ENSSSCG00000034741 | HOXD11 | 1.875530818 | up |
| ENSSSCG00000013731 | DNASE2 | 1.605882249 | up |
| Sus_scrofa_newGene_9450 | -- | 1.960090869 | up |
| ENSSSCG00000000216 | ASIC1 | 1.990386906 | up |
| ENSSSCG00000027762 | TNFRSF11B | -1.89663688 | down |
| ENSSSCG00000016322 | ACKR3 | -1.495600976 | down |
| Sus_scrofa_newGene_23295 | -- | -1.278789504 | down |
| ENSSSCG00000001418 | NEU1 | 1.223038022 | up |
| ENSSSCG00000013226 | PTPMT1 | 1.112681779 | up |
| ENSSSCG00000003081 | CEACAM16 | 1.865457143 | up |
| ENSSSCG00000016444 | TMEM176B | -1.294759795 | down |
| ENSSSCG00000010464 | PPP1R3C | -1.78553769 | down |
| ENSSSCG00000017068 | FAXDC2 | 2.100702415 | up |
| ENSSSCG00000013303 | ABTB2 | -1.489530381 | down |
| ENSSSCG00000001513 | SYNGAP1 | -1.306209744 | down |
| ENSSSCG00000007700 | HIP1 | -1.121951326 | down |
| ENSSSCG00000029425 | MRPS18B | 1.014090537 | up |
| ENSSSCG00000038671 | TNFRSF6B | -1.764427794 | down |
| ENSSSCG00000027426 | BCL3 | -1.03143951 | down |
| ENSSSCG00000017227 | FDXR | 1.7504569 | up |
| ENSSSCG00000002734 | MARVELD3 | 1.152041812 | up |
| Sus_scrofa_newGene_42159 | -- | 1.706657878 | up |
| ENSSSCG00000014233 | ENSSSCG00000014233 | -2.465847621 | down |
| ENSSSCG00000039442 | BMP2 | -1.644763633 | down |
| ENSSSCG00000007995 | MCRIP2 | 1.396474974 | up |
| ENSSSCG00000001963 | EGLN3 | -2.140804592 | down |
| ENSSSCG00000023666 | CTSB | 1.214304298 | up |
| ENSSSCG00000015774 | TENM3 | -1.945016178 | down |
| ENSSSCG00000020989 | BSND | 2.284078309 | up |
| ENSSSCG00000036437 | NOG | -2.541410876 | down |
| ENSSSCG00000011264 | CSRNP1 | -1.855163102 | down |
| Sus_scrofa_newGene_13635 | -- | 2.103825286 | up |
| Sus_scrofa_newGene_16153 | -- | 1.630655883 | up |
| ENSSSCG00000012026 | ADAMTS1 | -1.49989054 | down |
| ENSSSCG00000035074 | FOXO6 | -2.508120652 | down |
| ENSSSCG00000010102 | SLC7A4 | 1.229532242 | up |
| ENSSSCG00000014224 | SEMA6A | -1.314438802 | down |
| ENSSSCG00000024399 | EVC2 | -1.109985841 | down |
| ENSSSCG00000010114 | ESS2 | -3.412705507 | down |
| ENSSSCG00000011951 | NFKBIZ | -1.682638196 | down |
| ENSSSCG00000016991 | DUSP1 | -2.093331075 | down |
| ENSSSCG00000032778 | PLEKHG1 | -1.302573219 | down |
| ENSSSCG00000016473 | EPHB6 | 2.166367816 | up |
| ENSSSCG00000011133 | PFKFB3 | -2.098031605 | down |
| ENSSSCG00000001905 | ARID3B | -1.238390985 | down |
| Sus_scrofa_newGene_13579 | -- | -2.335506718 | down |
| ENSSSCG00000032081 | NDUFS3 | 1.310364161 | up |
| ENSSSCG00000000211 | AQP5 | 1.934992136 | up |
| ENSSSCG00000029066 | ENSSSCG00000029066 | 1.390046564 | up |
| ENSSSCG00000011623 | TPRA1 | 1.028164748 | up |
| ENSSSCG00000012771 | SLC6A8 | -1.41205203 | down |
| ENSSSCG00000017112 | IRX4 | 1.999523459 | up |
| ENSSSCG00000016851 | OSMR | -1.823182962 | down |
| ENSSSCG00000004458 | TPBG | -1.934553493 | down |
| ENSSSCG00000039798 | SLC11A1 | -1.313773774 | down |
| ENSSSCG00000040535 | ENSSSCG00000040535 | -1.733174175 | down |
| ENSSSCG00000007977 | HBM | -1.625905611 | down |
| ENSSSCG00000020701 | TTC9 | -1.448583911 | down |
| ENSSSCG00000013773 | ADGRL1 | -1.288874469 | down |
| Sus_scrofa_newGene_26847 | -- | 1.94403484 | up |
| Sus_scrofa_newGene_5918 | -- | -3.235694532 | down |
| ENSSSCG00000003807 | DNAJC6 | 1.193158533 | up |
| ENSSSCG00000015886 | ITGB6 | -2.065199544 | down |
| ENSSSCG00000036494 | ERG28 | 1.45056586 | up |
| Sus_scrofa_newGene_12618 | -- | 2.186361473 | up |
| ENSSSCG00000002929 | SYNE4 | 1.830002178 | up |
| ENSSSCG00000017251 | SOX9 | -2.355247358 | down |
| ENSSSCG00000030269 | ENSSSCG00000030269 | 2.034810738 | up |
| ENSSSCG00000034191 | SOX6 | -1.038798189 | down |
| ENSSSCG00000034338 | ECI1 | 1.075552249 | up |
| Sus_scrofa_newGene_38767 | -- | 1.771268483 | up |
| ENSSSCG00000006522 | GBA | 1.076511527 | up |
| ENSSSCG00000020895 | BICDL2 | 1.090077971 | up |
| ENSSSCG00000004718 | TTBK2 | -4.669614797 | down |
| ENSSSCG00000023187 | ENSSSCG00000023187 | -1.428643778 | down |
| ENSSSCG00000033702 | SBSN | -2.333913156 | down |
| ENSSSCG00000038500 | TRIB1 | -1.48609383 | down |
| ENSSSCG00000023706 | CHIA | -1.955845955 | down |
| ENSSSCG00000004952 | SMAD3 | -1.276211983 | down |
| ENSSSCG00000003805 | PDE4B | -1.56232862 | down |
| ENSSSCG00000036305 | ENSSSCG00000036305 | 1.150441407 | up |
| ENSSSCG00000039070 | ENSSSCG00000039070 | 1.781837396 | up |
| ENSSSCG00000037637 | GGH | 1.050461243 | up |
| ENSSSCG00000040581 | CISH | -2.00213978 | down |
| ENSSSCG00000017473 | TOP2A | -1.315763028 | down |
| ENSSSCG00000040569 | PLA2G10 | 1.761762276 | up |
| ENSSSCG00000025208 | RNF39 | -2.639989374 | down |
| ENSSSCG00000027249 | SLC25A2 | 1.301344746 | up |
| ENSSSCG00000031648 | WISP2 | -2.246039442 | down |
| ENSSSCG00000011643 | AMOTL2 | -1.197746752 | down |
| ENSSSCG00000009216 | SPP1 | -2.014095907 | down |
| ENSSSCG00000012967 | FOSL1 | -2.343457776 | down |
| ENSSSCG00000034609 | RNF112 | -2.392668134 | down |
| ENSSSCG00000039007 | CLEC4G | 1.829038411 | up |
| Sus_scrofa_newGene_42162 | -- | 2.393613195 | up |
| ENSSSCG00000015584 | PROX1 | -1.955238569 | down |
| ENSSSCG00000001750 | PAQR8 | 1.567486759 | up |
| ENSSSCG00000017906 | RNF167 | 1.242289201 | up |
| ENSSSCG00000000273 | ENSSSCG00000000273 | 1.073871274 | up |
| ENSSSCG00000040727 | ASPSCR1 | 3.051560291 | up |
| ENSSSCG00000004420 | TRAF3IP2 | -1.545188002 | down |
| ENSSSCG00000035984 | ENSSSCG00000035984 | -2.502842886 | down |
| ENSSSCG00000004789 | THBS1 | -2.194698994 | down |
| ENSSSCG00000030694 | PKP1 | -2.108837371 | down |
| ENSSSCG00000003684 | MTCL1 | -1.660178045 | down |
| ENSSSCG00000024666 | ENSSSCG00000024666 | 1.318481483 | up |
| ENSSSCG00000013554 | TRIP10 | -1.06556853 | down |
| ENSSSCG00000040977 | SNX8 | 1.073082946 | up |
| ENSSSCG00000026044 | FDFT1 | 1.242042198 | up |
| ENSSSCG00000004053 | TAGAP | -2.505235613 | down |
| ENSSSCG00000034500 | MSX2 | 1.331832706 | up |
| ENSSSCG00000038965 | ARC | -2.628650294 | down |
| ENSSSCG00000015426 | RELN | -1.882203569 | down |
| ENSSSCG00000005965 | MYC | -1.360289718 | down |
| ENSSSCG00000032843 | AHRR | -1.915584772 | down |
| ENSSSCG00000014156 | ARRDC3 | -1.129865937 | down |
| ENSSSCG00000016328 | RAB17 | 1.901477059 | up |
| ENSSSCG00000030680 | TCF7 | -1.453793128 | down |
| ENSSSCG00000005191 | MPDZ | -1.443588105 | down |
| ENSSSCG00000007436 | MMP9 | -1.765146427 | down |
| ENSSSCG00000000133 | TST | 1.541051556 | up |
| ENSSSCG00000003451 | ENSSSCG00000003451 | -2.145598094 | down |
| ENSSSCG00000012625 | PGRMC1 | -1.483564375 | down |
| ENSSSCG00000022584 | PPP1R3F | 1.777867146 | up |
| ENSSSCG00000034282 | ABCA12 | -2.344142424 | down |
| ENSSSCG00000002937 | ZNF420 | -1.216540983 | down |
| ENSSSCG00000004132 | PHACTR2 | -1.140498488 | down |
| Sus_scrofa_newGene_41415 | -- | 1.995115813 | up |
| ENSSSCG00000025414 | PYY | -2.28691944 | down |
| ENSSSCG00000008678 | TMEM129 | 1.183679897 | up |
| ENSSSCG00000004369 | PRDM1 | -2.290447314 | down |
| Sus_scrofa_newGene_40920 | -- | 1.841363302 | up |
| ENSSSCG00000004490 | SETBP1 | -1.325961803 | down |
| ENSSSCG00000040689 | APOA4 | -2.798898742 | down |
| ENSSSCG00000001488 | GCLC | 1.153081621 | up |
| ENSSSCG00000012309 | SHROOM4 | -1.133830546 | down |
| ENSSSCG00000035836 | DDT | 1.120509023 | up |
| ENSSSCG00000011968 | NIT2 | 1.057068571 | up |
| ENSSSCG00000016487 | MGAM2 | -2.33957151 | down |
| ENSSSCG00000037754 | SLC39A11 | 1.418259044 | up |
| ENSSSCG00000035945 | FDX1 | 1.479709589 | up |
| ENSSSCG00000010853 | EPHX1 | 1.676764086 | up |
| ENSSSCG00000003755 | MCOLN2 | 2.113820513 | up |
| ENSSSCG00000001787 | IL16 | -1.310552967 | down |
| ENSSSCG00000033760 | LTBP4 | -1.322932165 | down |
| ENSSSCG00000000705 | CD27 | 2.047716414 | up |
| ENSSSCG00000035324 | EN2 | 1.71530051 | up |
| ENSSSCG00000016676 | INMT | 1.942865715 | up |
| ENSSSCG00000002386 | IFT43 | -1.067929196 | down |
| ENSSSCG00000030626 | ALDH1L1 | 1.745446024 | up |
| ENSSSCG00000040673 | TMEM140 | 1.28237381 | up |
| ENSSSCG00000011195 | GALNT15 | -1.612741657 | down |
| ENSSSCG00000022246 | ENSSSCG00000022246 | -1.425313038 | down |
| ENSSSCG00000023749 | MIOX | 1.57581602 | up |
| ENSSSCG00000001636 | GUCA1A | -2.2839451 | down |
| ENSSSCG00000027525 | DHCR24 | 1.286415971 | up |
| ENSSSCG00000027646 | TIPARP | -1.916380991 | down |
| ENSSSCG00000010461 | ANKRD1 | -2.640060937 | down |
| ENSSSCG00000007737 | TPST1 | 1.075489833 | up |
| Sus_scrofa_newGene_44975 | -- | -2.211311177 | down |
| ENSSSCG00000005610 | SLC2A8 | 1.598154728 | up |
| ENSSSCG00000021597 | PHLDA2 | -1.283222245 | down |
| ENSSSCG00000002818 | PLLP | 1.424996708 | up |
| ENSSSCG00000024103 | ADPRHL1 | -2.488271094 | down |
| ENSSSCG00000035774 | ERRFI1 | -3.153823529 | down |
| ENSSSCG00000032768 | EBP | 1.482665013 | up |
| ENSSSCG00000023141 | RNLS | 1.289429421 | up |
| ENSSSCG00000037065 | EMP2 | -1.210994462 | down |
| ENSSSCG00000032320 | C8orf4 | -1.204546539 | down |
| ENSSSCG00000007522 | CTSZ | 1.323323393 | up |
| ENSSSCG00000031592 | ZNF462 | -1.542616195 | down |
| ENSSSCG00000022635 | BEX4 | 1.612794779 | up |
| ENSSSCG00000001081 | SOX4 | -1.279134238 | down |
| ENSSSCG00000030502 | CEP170 | -1.011187813 | down |
| Sus_scrofa_newGene_37141 | -- | -2.408773024 | down |
| ENSSSCG00000006286 | ENSSSCG00000006286 | -1.441440874 | down |
| ENSSSCG00000006731 | VTCN1 | -1.772649147 | down |
| ENSSSCG00000008034 | NOXO1 | 1.853389405 | up |
| ENSSSCG00000030113 | SHISA2 | -2.036145694 | down |
| Sus_scrofa_newGene_21175 | -- | 2.15563848 | up |
| ENSSSCG00000013269 | MAPK8IP1 | 1.584677291 | up |
| ENSSSCG00000035077 | INHBA | -2.25619456 | down |
| ENSSSCG00000010116 | SLC25A1 | 1.30484555 | up |
| ENSSSCG00000010604 | SH3PXD2A | -1.827521058 | down |
| ENSSSCG00000022140 | TMPRSS11E | -2.253297773 | down |
| ENSSSCG00000023525 | TMEM26 | -1.728108244 | down |
| ENSSSCG00000007034 | PSD3 | -1.803529791 | down |
| ENSSSCG00000017403 | STAT3 | -1.050465988 | down |
| ENSSSCG00000012854 | RASSF7 | 1.092725946 | up |
| ENSSSCG00000006793 | ENSSSCG00000006793 | 1.160742256 | up |
| ENSSSCG00000012151 | NHS | -1.615286927 | down |
| ENSSSCG00000014960 | AMOTL1 | -1.256248629 | down |
| ENSSSCG00000034863 | PARD6A | 2.406933711 | up |
| ENSSSCG00000036975 | PSRC1 | 1.342137465 | up |
| ENSSSCG00000038801 | NPNT | 1.538602412 | up |
| ENSSSCG00000000991 | FOXQ1 | -2.13675867 | down |
| ENSSSCG00000015954 | DLX2 | 1.156751763 | up |
| ENSSSCG00000039425 | BPGM | 1.129736384 | up |
| ENSSSCG00000022401 | AGTRAP | 1.158410484 | up |
| ENSSSCG00000000728 | PARP11 | -1.238428018 | down |
| Sus_scrofa_newGene_10848 | -- | 1.680745974 | up |
| ENSSSCG00000000901 | FGD6 | -1.336138402 | down |
| ENSSSCG00000016678 | NOD1 | -2.246219194 | down |
| ENSSSCG00000023322 | COL9A2 | -1.613360503 | down |
| ENSSSCG00000004089 | RMND1 | 1.091096346 | up |
| Sus_scrofa_newGene_31815 | -- | 2.935723419 | up |
| ENSSSCG00000015677 | LYPD6B | 1.69910232 | up |
| ENSSSCG00000040260 | ENSSSCG00000040260 | 1.728929616 | up |
| Sus_scrofa_newGene_22748 | -- | -4.362519825 | down |
| ENSSSCG00000003928 | PLK3 | -1.873654457 | down |
| ENSSSCG00000022839 | ENSSSCG00000022839 | -2.160827666 | down |
| ENSSSCG00000010742 | UROS | 1.607426207 | up |
| ENSSSCG00000024336 | TMPRSS2 | 1.399665165 | up |
| ENSSSCG00000032434 | PLAUR | -1.972305454 | down |
| ENSSSCG00000026092 | SREBF2 | 1.378130355 | up |
| Sus_scrofa_newGene_41945 | -- | 2.552021518 | up |
| ENSSSCG00000003000 | ITPKC | -1.732283361 | down |
| Sus_scrofa_newGene_10126 | -- | 1.599401677 | up |
| ENSSSCG00000002901 | UPK1A | 2.07760151 | up |
| ENSSSCG00000029519 | CIAPIN1 | 1.082897848 | up |
| ENSSSCG00000004192 | CTGF | -1.480920916 | down |
| ENSSSCG00000007978 | ENSSSCG00000007978 | -1.532199337 | down |
| ENSSSCG00000006873 | FRRS1 | 1.155488724 | up |
| ENSSSCG00000032591 | ENSSSCG00000032591 | -2.508878103 | down |
| ENSSSCG00000005287 | PSAT1 | 1.804479479 | up |
| ENSSSCG00000014401 | NR3C1 | -1.377036922 | down |
| ENSSSCG00000002690 | GAN | -1.420835282 | down |
| ENSSSCG00000010428 | DKK1 | -3.35608602 | down |
| ENSSSCG00000017791 | SSH2 | -1.323255052 | down |
| ENSSSCG00000005852 | ENTPD8 | 1.700099728 | up |
| ENSSSCG00000022780 | UGCG | -1.244544541 | down |
| ENSSSCG00000024697 | TMEM125 | 1.090845047 | up |
| ENSSSCG00000003951 | C1orf210 | 1.589037912 | up |
| ENSSSCG00000037878 | AHSP | -1.530229527 | down |
| ENSSSCG00000011453 | ITIH4 | -1.699496784 | down |
| ENSSSCG00000011630 | ACKR4 | -2.292145925 | down |
| ENSSSCG00000012523 | ENSSSCG00000012523 | 1.15619328 | up |
| ENSSSCG00000010703 | ENSSSCG00000010703 | -1.900465107 | down |
| ENSSSCG00000016892 | FST | -1.054265853 | down |
| ENSSSCG00000009844 | HSPB8 | -2.15887998 | down |
| ENSSSCG00000012152 | SCML1 | 1.199415331 | up |
| ENSSSCG00000006194 | NCOA2 | 1.053446498 | up |
| ENSSSCG00000009290 | MIPEP | 1.023170431 | up |
| ENSSSCG00000002496 | GLRX5 | 1.59934378 | up |
| Sus_scrofa_newGene_41744 | -- | 1.344969684 | up |
| ENSSSCG00000022636 | DENND5B | -1.649789314 | down |
| ENSSSCG00000012525 | BEX1 | 1.270278545 | up |
| ENSSSCG00000013408 | ADM | -1.675401794 | down |
| ENSSSCG00000007864 | GPRC5B | 1.437890633 | up |
| ENSSSCG00000037935 | CYYR1 | -1.121534271 | down |
| ENSSSCG00000022236 | FOLR1 | 1.816558448 | up |
| ENSSSCG00000015982 | HOXD9 | 1.343786404 | up |
| ENSSSCG00000021371 | ZNF300 | -1.384608944 | down |
| Sus_scrofa_newGene_2086 | -- | 1.954781528 | up |
| ENSSSCG00000001710 | RUNX2 | -2.465030467 | down |
| Sus_scrofa_newGene_38554 | -- | 1.584061212 | up |
| ENSSSCG00000017184 | PRPSAP1 | 1.28939037 | up |
| ENSSSCG00000016591 | PAX4 | 1.828767381 | up |
| ENSSSCG00000021355 | SOD1 | 1.151036607 | up |
| ENSSSCG00000012303 | PAGE4 | 1.455084583 | up |
| ENSSSCG00000034763 | IRS2 | -1.784052662 | down |
| ENSSSCG00000007032 | PLAT | -2.013528612 | down |
| ENSSSCG00000030696 | SLC19A1 | 1.657666424 | up |
| ENSSSCG00000000135 | KCTD17 | 1.501017334 | up |
| ENSSSCG00000024958 | GPR173 | -1.126327983 | down |
| ENSSSCG00000006651 | ADAMTSL4 | -1.364587636 | down |
| ENSSSCG00000004195 | ARG1 | -1.995799314 | down |
| ENSSSCG00000007727 | AUTS2 | -1.825763498 | down |
| ENSSSCG00000012583 | ACSL4 | -2.18942461 | down |
| ENSSSCG00000014985 | MMP3 | -2.291053625 | down |
| ENSSSCG00000034720 | IQCK | 1.517558685 | up |
| ENSSSCG00000009317 | CDX2 | 1.919489225 | up |
| ENSSSCG00000013933 | PBX4 | 1.330538369 | up |
| ENSSSCG00000006105 | GEM | -2.068855453 | down |
| ENSSSCG00000011218 | SLC4A7 | -1.42855482 | down |
| ENSSSCG00000005166 | MLLT3 | -1.438155494 | down |
| ENSSSCG00000022017 | LGALS4 | 1.559245052 | up |
| ENSSSCG00000010791 | FUOM | 5.816652486 | up |
| ENSSSCG00000008953 | IL8 | -2.422923053 | down |
| ENSSSCG00000005045 | BMP4 | -1.163575967 | down |
| ENSSSCG00000015981 | HOXD10 | 1.661923912 | up |
| ENSSSCG00000016431 | GALNTL5 | 3.112370802 | up |
| ENSSSCG00000001431 | ENSSSCG00000001431 | 1.674583129 | up |
| ENSSSCG00000009348 | STARD13 | -1.553803235 | down |
| ENSSSCG00000038838 | DLX5 | 1.966718673 | up |
| ENSSSCG00000007493 | ENSSSCG00000007493 | 1.369085518 | up |
| ENSSSCG00000007192 | C20orf202 | 1.874577845 | up |
| ENSSSCG00000038494 | LPIN2 | -1.459675092 | down |
| ENSSSCG00000013360 | TMEM86A | 1.805225928 | up |
| ENSSSCG00000009592 | NFIL3 | -2.276479998 | down |
| ENSSSCG00000007027 | SLC20A2 | -1.529915336 | down |
| ENSSSCG00000011141 | CALM | 2.235342443 | up |
| ENSSSCG00000003761 | ADGRL2 | -1.373152768 | down |
| ENSSSCG00000037501 | FANCF | 1.295859142 | up |
| ENSSSCG00000025826 | BOC | -1.021966024 | down |
| ENSSSCG00000014818 | STARD10 | 1.040263557 | up |
| ENSSSCG00000020970 | IL6 | -2.869406076 | down |
| ENSSSCG00000016210 | ABCB6 | 1.336098036 | up |
| ENSSSCG00000012840 | CD151 | -1.431107494 | down |
| ENSSSCG00000005222 | SLC1A1 | 2.116522946 | up |
| ENSSSCG00000012347 | ALAS2 | -1.746829875 | down |
| ENSSSCG00000016873 | NIM1K | -1.459867891 | down |
| ENSSSCG00000040445 | RND3 | -1.378152825 | down |
| ENSSSCG00000027030 | BDKRB2 | -1.670621086 | down |
| ENSSSCG00000017879 | SPNS2 | -1.885473809 | down |
| ENSSSCG00000022638 | ATP12A | 1.694565914 | up |
| ENSSSCG00000023426 | ENSSSCG00000023426 | 1.337879811 | up |
| ENSSSCG00000036956 | ENSSSCG00000036956 | -1.421502809 | down |
| ENSSSCG00000014927 | NOX4 | -1.441094401 | down |
| ENSSSCG00000010325 | KCNMA1 | -2.177464748 | down |
| ENSSSCG00000005385 | NR4A3 | -1.576305634 | down |
| ENSSSCG00000007481 | ZFP64 | 1.482732091 | up |
| ENSSSCG00000017087 | GM2A | 1.284940376 | up |
| ENSSSCG00000011485 | PTPRG | -1.150870521 | down |
| ENSSSCG00000024428 | CHRNA9 | 1.39895227 | up |
| ENSSSCG00000033337 | ARHGDIB | 1.209233028 | up |
| ENSSSCG00000004807 | SCG5 | -2.059657548 | down |
| ENSSSCG00000006982 | ZDHHC2 | -1.736157701 | down |
| ENSSSCG00000024018 | SLC16A3 | -1.572696607 | down |
| ENSSSCG00000011880 | EAF2 | 1.143949975 | up |
| ENSSSCG00000003402 | PGD | 1.014671119 | up |
| ENSSSCG00000038192 | ENSSSCG00000038192 | -1.908269806 | down |
| ENSSSCG00000012029 | BACH1 | -1.053394273 | down |
| ENSSSCG00000037241 | RGS2 | -1.644302472 | down |
| ENSSSCG00000003079 | PVR | -1.194381744 | down |
| ENSSSCG00000012027 | ADAMTS5 | -1.897438512 | down |
| ENSSSCG00000023186 | CA4 | 2.062005752 | up |
| ENSSSCG00000017018 | TENM2 | 2.361089788 | up |
| ENSSSCG00000010332 | PLAC9 | -1.455340514 | down |
| ENSSSCG00000031378 | KBTBD6 | 1.718276006 | up |
| ENSSSCG00000038783 | IGFBP3 | -1.811645287 | down |
| ENSSSCG00000039523 | ENSSSCG00000039523 | 1.184186627 | up |
| ENSSSCG00000036377 | OVOL2 | 1.652150537 | up |
| ENSSSCG00000037102 | FSTL3 | -1.446071052 | down |
| ENSSSCG00000004441 | DSE | -1.457344588 | down |
| ENSSSCG00000040725 | IL11 | -1.753497849 | down |
| ENSSSCG00000016671 | CCDC129 | 1.418327778 | up |
| ENSSSCG00000001549 | FKBP5 | -1.488885175 | down |
| ENSSSCG00000017865 | CTNS | 1.18295514 | up |
| ENSSSCG00000017640 | RNF43 | 1.034526061 | up |
| ENSSSCG00000014725 | HBB | -1.891522045 | down |
| ENSSSCG00000013147 | FAM111B | -1.579911333 | down |
| Sus_scrofa_newGene_5167 | -- | 2.0542277 | up |
| ENSSSCG00000032341 | AIF1 | 1.309525939 | up |
| ENSSSCG00000006940 | CCN1 | -1.382165798 | down |
| ENSSSCG00000007043 | GPCPD1 | -1.036007975 | down |
| ENSSSCG00000025698 | SERPINE1 | -2.296619616 | down |
| ENSSSCG00000029815 | SRGAP1 | -1.102295857 | down |
| ENSSSCG00000038180 | ADAMTS3 | -2.151099556 | down |
| ENSSSCG00000004222 | NCOA7 | -1.132166943 | down |
| ENSSSCG00000036027 | TPRN | 1.711347729 | up |
| ENSSSCG00000005533 | PTGS1 | -1.514347591 | down |
| ENSSSCG00000008326 | TGFA | -3.517415394 | down |
| ENSSSCG00000036060 | RRAD | -1.640253018 | down |
| Sus_scrofa_newGene_52751 | -- | 1.977992586 | up |
| ENSSSCG00000021911 | NDRG4 | -1.845852006 | down |
| ENSSSCG00000035335 | FGL1 | 2.168491681 | up |
| ENSSSCG00000018046 | ENSSSCG00000018046 | 1.82162471 | up |
| ENSSSCG00000000802 | NELL2 | -2.272462125 | down |
| ENSSSCG00000001620 | MDFI | -1.869488131 | down |
| ENSSSCG00000034905 | MVD | 1.617004254 | up |
| ENSSSCG00000032996 | SLC7A5 | -1.888002219 | down |
| ENSSSCG00000005036 | GPR137C | 1.863555964 | up |
| ENSSSCG00000011350 | CCDC51 | 1.309266256 | up |
| ENSSSCG00000035037 | ENSSSCG00000035037 | -1.866868771 | down |
| ENSSSCG00000024389 | SIGIRR | 1.221709947 | up |
| ENSSSCG00000021181 | DHCR7 | 1.475315798 | up |
| ENSSSCG00000016430 | GALNT11 | 1.395100365 | up |
| ENSSSCG00000033919 | DCLK1 | -1.797562573 | down |
| Sus_scrofa_newGene_10074 | -- | -2.246247099 | down |
| ENSSSCG00000031903 | TNNT3 | -2.377357252 | down |
| ENSSSCG00000011583 | TMEM40 | -1.525844422 | down |
| ENSSSCG00000038730 | SSFA2 | -1.346265399 | down |
| ENSSSCG00000036213 | FGF2 | -2.2318817 | down |
| ENSSSCG00000040348 | LIN7A | -1.516310494 | down |
| ENSSSCG00000024439 | PTGER4 | -2.463200421 | down |
| ENSSSCG00000017755 | NOS2 | 1.812348555 | up |
| ENSSSCG00000038439 | PRX | 1.231655307 | up |
| ENSSSCG00000014362 | HBEGF | -1.609676019 | down |
| ENSSSCG00000036723 | EMP1 | -1.781294304 | down |
| ENSSSCG00000036534 | MYOM3 | -2.255641195 | down |
| ENSSSCG00000032527 | FOSL2 | -1.578833177 | down |
| Sus_scrofa_newGene_31399 | -- | -1.977733456 | down |
| ENSSSCG00000000377 | ENSSSCG00000000377 | -2.504133185 | down |
| ENSSSCG00000012234 | SRPX | -1.222251178 | down |
| ENSSSCG00000030998 | WIF1 | -3.097091993 | down |
| ENSSSCG00000031456 | ARL5B | -1.050215197 | down |
| ENSSSCG00000027745 | ABCG1 | -1.267240446 | down |
| ENSSSCG00000034378 | IFNGR2 | -1.083407288 | down |
| ENSSSCG00000015100 | UPK2 | 1.347366261 | up |
| ENSSSCG00000005593 | OLFML2A | -1.194753442 | down |
| ENSSSCG00000001639 | TRERF1 | -1.295247014 | down |
| ENSSSCG00000039351 | PIGH | 1.505863173 | up |
| ENSSSCG00000026819 | NID1 | -1.543634611 | down |
| ENSSSCG00000001927 | ENSSSCG00000001927 | 1.457005752 | up |
| ENSSSCG00000017203 | GALK1 | 1.248473342 | up |
| ENSSSCG00000003325 | ENSSSCG00000003325 | -1.066768621 | down |
| ENSSSCG00000028294 | CATSPER3 | 1.504947986 | up |
| ENSSSCG00000012633 | IL13RA1 | -1.265341553 | down |
| Sus_scrofa_newGene_10148 | -- | 2.422913514 | up |
| ENSSSCG00000016578 | FLNC | -2.326820742 | down |
| ENSSSCG00000027652 | KLHL15 | -1.007269226 | down |
| ENSSSCG00000012409 | ENSSSCG00000012409 | 1.615581183 | up |
| ENSSSCG00000009281 | SGCG | -1.002617815 | down |
| ENSSSCG00000039847 | C1S | -1.008119301 | down |
| ENSSSCG00000016557 | CPA1 | 2.405565958 | up |
| ENSSSCG00000009413 | CPB2 | 1.701804758 | up |
| ENSSSCG00000008812 | ATP10D | -1.035647789 | down |
| ENSSSCG00000015930 | DHRS9 | -1.658043936 | down |
| ENSSSCG00000036724 | CRYAB | -2.085784163 | down |
| ENSSSCG00000008959 | CXCL2 | -1.777721232 | down |
| ENSSSCG00000023584 | ENSSSCG00000023584 | 2.096683152 | up |
| ENSSSCG00000016665 | BMPER | -1.053484427 | down |
| Sus_scrofa_newGene_55704 | -- | -1.549643951 | down |
| ENSSSCG00000035908 | ENSSSCG00000035908 | 1.421166311 | up |
